# The Causal Association of Irritable Bowel Syndrome with Multiple Disease Outcomes: A Phenome-Wide Mendelian Randomization Study

**DOI:** 10.3390/jcm12031106

**Published:** 2023-01-31

**Authors:** Chunyang Li, Yilong Chen, Yi Chen, Zhiye Ying, Yao Hu, Yalan Kuang, Huazhen Yang, Huan Song, Xiaoxi Zeng

**Affiliations:** 1 Biomedical Big Data Center, West China Hospital, West China School of Medicine, Sichuan University, 37 Guo Xue Xiang, Chengdu 610041, China; 2 Med-X Center for Informatics, Sichuan University, 17 Ren Min Nan Road 3rd Section, Chengdu 610041, China; 3 Department of Gastrointestinal Surgery, West China Hospital, West China School of Medicine, Sichuan University, 37 Guo Xue Xiang, Chengdu 610041, China

**Keywords:** irritable bowel syndrome, phenome-wide association study, individual-level Mendelian randomization, summary-level Mendelian randomization

## Abstract

Background: This study aimed to identify novel associations between irritable bowel syndrome (IBS) and a broad range of outcomes. Methods: In total, 346,352 white participants in the U.K. Biobank were randomly divided into two halves, in which a genome-wide association study (GWAS) of IBS and a polygenic risk score (PRS) analysis of IBS using GWAS summary statistics were conducted, respectively. A phenome-wide association study (PheWAS) based on the PRS of IBS was performed to identify disease outcomes associated with IBS. Then, the causalities of these associations were tested by both one-sample (individual-level data in U.K. Biobank) and two-sample (publicly available summary statistics) Mendelian randomization (MR). Sex-stratified PheWAS-MR analyses were performed in male and female, separately. Results: Our PheWAS identified five diseases associated with genetically predicted IBS. Conventional MR confirmed these causal associations between IBS and depression (OR: 1.07, 95%CI: 1.01–1.14, *p* = 0.02), diverticular diseases of the intestine (OR: 1.13, 95%CI: 1.08–1.19, *p* = 3.00 × 10^−6^), gastro-esophageal reflux disease (OR: 1.09, 95%CI: 1.05–1.13, *p* = 3.72 × 10^−5^), dyspepsia (OR: 1.21, 95%CI: 1.13–1.30, *p* = 9.28 × 10^−8^), and diaphragmatic hernia (OR: 1.10, 95%CI: 1.05–1.15, *p* = 2.75 × 10^−5^). The causality of these associations was observed in female only, but not men. Conclusions: Increased risks of IBS is found to cause a series of disease outcomes. Our findings support further investigation on the clinical relevance of increased IBS risks with mental and digestive disorders.

## 1. Introduction

Irritable bowel syndrome (IBS) is a common disorder worldwide and is characterized by recurrent abdominal pain related to defecation or accompanied by a change in habit frequency or form of stool, according to the criteria of Rome IV [1]. The prevalence of IBS is high in Western countries, and in the general population, it is more common in women [2] and young adults [3]. IBS significantly reduces health-related quality of life and accounts for a considerable economic burden [4].

Previous observational studies have identified that IBS associated with glaucoma [5], osteoporosis [6], Parkinson’s disease [7], anxiety [8], depression [8], neuroticism [9], migraine [10], asthma [11], a gluten-containing diet [12], insomnia [13], some organic gastrointestinal diseases [14], and functional gastrointestinal disorders [15]. However, results from the above-mentioned observational studies may be biased by potential confounding factors that have not been fully accounted for. Emerging evidence suggests that there may be underlying pathophysiological disturbances and that IBS is unlikely to be merely a somatosensory disorder [1]. Furthermore, research indicates that sex hormones play a role in the pathophysiology of IBS [16]; however, no studies have compared the differences in the broad-spectrum of health conditions associated with IBS between males and females.

A phenome-wide association study (PheWAS) together with Mendelian randomization (MR), using genetic liability as a proxy for phenotypic measures, enables an unbiased and hypothesis-free scan through a wide range of phenotypes and systematically analyzes causal inferences. MR utilizes genetic variants as instrumental variables to approximate the lifetime status of an exposure (herein IBS) and evaluates its causal effect on a clinical outcome, which could overcome the limitations of observational studies susceptible to reverse causality and residual confounding. MR leverages the random assignment of genetic variants independent of environmental confounders; therefore, causal estimates of exposure risks are substantially less confounded and not susceptible to reverse causality.

To comprehensively validate any possible health effects of IBS, in this study, we conducted PheWAS to identify novel associations between genetically predicted IBS and a broad range of outcomes in the U.K. Biobank, which is a large, population-based cohort. To evaluate the influence of sex, we performed sex-stratified analyses in male and female subjects, separately. Furthermore, we tested for causality of these phenome-wide associations using both one-sample (based on individual-level data) and two-sample (based on summary Genome-wide association study [GWAS] statistics) MR.

## 2. Methods

### 2.1. Participants

In the current study, the participants were recruited from the U.K. Biobank. Briefly, the U.K. Biobank recruited nearly half a million participants aged 40–69 years from the United Kingdom (U.K.) general population between 2006 and 2010. At the baseline assessment, data on participants’ medical history, demographic information, and lifestyle were collected. The participants were then followed up, and health-related outcomes were obtained through linkage to medical and other health-related records, including the Hospital Episode Statistics (HES) for England, Scottish Morbidity Record, Patient Episode Database for Wales, National Health Service (NHS) Digital for England and Wales, NHS Central Register for Scotland, and patient records from general health care practitioners.

**Figure 1:**
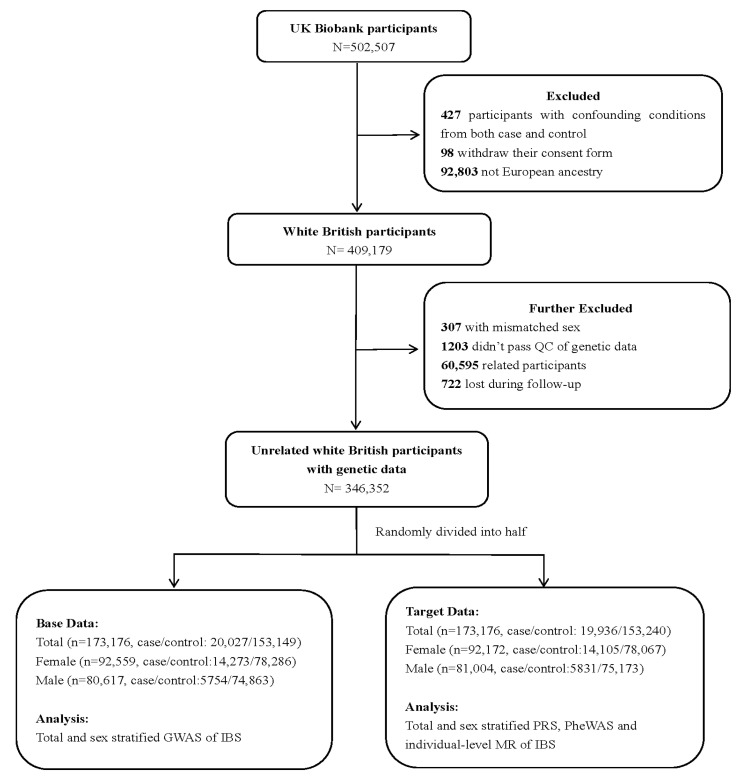
The flow of participants through the study. QC—quality control; GWAS—genome-wide association study; IBS—irritable bowel syndrome; PRS—polygenic risk score; PheWAS—phenome-wide association study; MR—Mendelian randomization.

IBS cases were identified according to the following four alternative criteria [9]: (1) fulfilling Roma III symptom criteria for IBS; (2) answered “yes” to the question “Have you ever been diagnosed with IBS ?” in Digestive Health Questionnaire (DHQ); (3) self-reported IBS diagnosis; and (4) a primary or secondary clinical International Classification of Diseases version 10 (ICD-10) diagnosis of IBS (Supplementary Methods). In this study, patients with other confounding medical conditions, such as inflammatory bowel disease or previous intestinal resectional surgery before IBS diagnosis, were excluded (*n* = 427) (coding for exclusion criteria are listed in Table S1). We further excluded U.K. Biobank participants who withdrew their consent (*n* = 98), those were not European ancestry (*n* = 92,803), those without genetic data, those who did not pass the quality control of genetic data (i.e., mismatch sex, high rate of missingness and heterozygosity, kinship coefficient over 0.0884, *n* = 62,105), and those who were lost to follow-up (*n* = 722). Following the application of the exclusion criteria, 346,352 unrelated white British individuals were included in our final analysis (Figure 1).

We followed the Strengthening the Reporting of Observational Studies in Epidemiology (STROBE) reporting guideline.

### 2.2. Phenome-Wide Association Study

Prior to PheWAS, we performed GWAS for IBS in the U.K. Biobank and calculated the polygenic risk score (PRS) for each participant as a proxy of genetic liability for IBS. We randomly divided the study population into base (*n* = 173,176; case/control: 20,027/153,149) and target (*n* = 173,176; case/control:19,936/153,240) samples to avoid sample overlap (Figure 1), as required by the PRS calculation [17]. After the standard quality control procedure, a generalized linear mixed model (GLMM), controlling for population stratification and relatedness, was adopted for the GWAS in base data (Supplementary Methods) [18]. Then, the PRS of IBS derived from 58,314 single nucleotide polymorphisms (SNPs) based on the above-mentioned GWAS was calculated using PLINK1.9 software in the target data (Supplementary Methods) [17].

The phenome codes used in our study were derived from the primary and secondary diagnoses of ICD-10 and the International Classification of Diseases version 9 (ICD-9) codes in the HES in the U.K. Biobank. The inpatient diagnoses of the U.K. Biobank adopted predominantly the ICD-10 disease classification and, in the early years, a small fraction of the ICD-9 coding system, which we firstly converted to the corresponding ICD-10 codes using general equivalence mappings [19] and manual curation [20]. Then, we used the 3-digit ICD-10 codes as phecodes to identify medical conditions by combining diagnoses with the same first three ICD-10 codes. Subsequently, we excluded phecodes related to perinatal conditions, unclassified symptoms or signs, injuries, poisoning, morbidity, and mortality (Restricted in Chapter 1–15 and 17 of ICD-10).

We maintained only phecodes with more than 250 cases [21]. Thus, 665 phecodes were included in the study (the basic statistics of the phenotypes and their case numbers are shown in Table S2). We explored the association between the scaled PRS of IBS and each phenotype with logistic regression in target data, adjusting for age (continuous variables), sex (male/female), assessment cancer (category variables), and the first 10 genetic principal components (PCs) (continuous variables). PRS of IBS was scaled to a unit reflecting one SD (equals to 1) increase of PRS. We applied the Bonferroni correction (P threshold = 0.05/665 phecodes = 7.52 × 10^−5^) to correct for multiple testing.

### 2.3. Mendelian Randomization

Individual- and summary-level MRs were performed to test the causal associations between IBS and disease outcomes identified in the PheWAS analysis.

#### 2.3.1. Individual-Level MR for Causal Association between IBS with Health Outcomes

Individual-level MR was performed for target data by using two-stage sequential regression analysis: the first-stage regressed the exposure (scaled PRS of IBS) on the outcome of IBS, and the second-stage regressed the fitted values of the exposure from the first stage on the outcome. Both stages were adjusted for age (continuous variable), sex (male/female), and the first 10 genetic PCs (continuous variable).

#### 2.3.2. Summary-Level MR for Causal Association between IBS with Health Outcomes

In order to increase statistical power, genetic instruments for exposure were derived from a previously published study [9]. Instrumental variables were selected according to the following criteria: (1) independent SNPs with a linkage disequilibrium threshold r^2^ < 0.001 within a clumping distance of 10,000 kb. Clumping was performed using PLINK1.9 with the 1000 Genomes project as the reference panel. (2) Significant exposure with a cut-off at 1 × 10^−5^. In total, 80 SNPs were used as genetic instruments (Table S3). The corresponding *F*-statistics were 19 to 39, indicating sufficient strength of instrumental variables (IVs) [22].

For the health outcomes, corresponding variant–outcome effects were estimated from the total 346,452 unrelated white British participants from the U.K. Biobank with genetic data using logistic regression, as previously described.

We firstly harmonized IVs-exposure and IVs-outcome, and IVs with intermediate allele frequency (i.e., 0.42–0.58) were excluded from MR analysis. The inverse-variance weighted (IVW) model was estimated by the ratio method, in which the ratios of the beta coefficient of the SNP-outcome association divided by that of the SNP-exposure biomarker association were calculated and then meta-analyzed [23]. To further address the potential directional pleiotropic effects, we conducted MR-Egger regression, detecting potential pleiotropy by allowing a non-zero intercept term, with P intercept < 0.05 indicating possible pleiotropy [24]. Herein, MR IVW and MR Egger were selected as primary analysis.

#### 2.3.3. Sensitivity Analysis

A valid genetic instrumental variable used in MR analysis should satisfy all of the following three assumptions: (1) significantly associated with exposure, (2) not associated with outcome via a confounding pathway, and (3) not affecting the outcome directly via exposure. The second and third assumptions for IVs cannot be easily tested, we performed several sensitivity analyses to account for potential bias in evaluating the robustness of the MR results.

Firstly, we performed several analyses to test the robustness of the causal associations between IBS and health outcomes. First (1), we used alternative median- or mode-based weighted methods, which allow some invalid IVs and increase robustness when pleiotropy exists [25]. Usually, it is not used as a standard for causal estimators because of its low power, but the same direction resulting from weighted median and mode methods consistent with IVW results can be viewed as additional supporting evidence. Second (2), we evaluated potential heterogeneity across genetic instruments with Cochran’s Q test. Third (3), MR Pleiotropy Residual Sum and Outlier (MR PRESSO) was performed. Potential heterogeneity among IVs was also tested by using the MR PRESSO global test. Significant outliers were detected by the MR PRESSO outlier test and removed, and the MR PRESSO distortion test was performed to detect differences after removing outliers [26]. Fourth (4), the MR Steiger directionality test was performed to compare the instruments’ association with the exposure and outcome to determine whether there is evidence that the assumed direction of causality is correct. Fifth (5), visually leave-one-out and scatter plots were performed to confirm that the results were not driven by a specific genetic instrument. Finally (6), we manually pruned pleiotropic SNPs, which were significantly associated (*p* < 7.52 × 10^−5^, 0.05/665) with risk factors of IBS and outcomes identified in PheWAS analysis, and re-ran the primary MR analysis.

Secondly, to replicate our findings on the association between IBS and disease outcomes in the U.K. Biobank dataset, we replicated the MR analysis using publicly available summary-level data for depression, gastro-esophageal reflux disease (GERD), dyspepsia, diaphragmatic hernia, and diverticular disease as outcomes and publicly available IBS GWAS summary data as exposure data. (Supplementary Methods).

Reversed MR was performed to identify whether there was a reverse causality between the outcomes identified and IBS. Publicly available IBS GWAS summary data were used as the outcomes. Other diseases were used as exposures.

### 2.4. Sex-Stratification Analysis

To evaluate the influence of sex on the associated IBS disease spectrum, PheWAS-MR analyses as described above were performed separately for female and male participants. We first performed GWAS using GLMM in 92,559 female and 80,617 male participants from the base data (Figure 1). Then, 57,954 SNPs for female and 163,411 SNPs for male were retained for sex-specific PRS analyses (Supplementary Methods). Sex-stratified PheWAS and individual-level MR analyses based on scaled PRS were performed according to the analysis procedure described above. There are no publicly available sex-stratified GWAS summary data for IBS; therefore, we did not perform summary-level MR.

The original estimate of MR (β) corresponds to the change in log odds for disease outcome per unit increase in log odds of IBS and was further scaled to per doubling in the odds of IBS by multiplying by 0.693 [27]. In the current study, we estimated that we had 80% power to detect a 50% and 80% increase in risk per genetically determined IBS increase for 37 and 106 outcomes with α = 0.05 and r^2^ = 0.0012 (Table S4), using the method proposed by Burgess [28]. Data were analyzed using the “TwoSampleMR” package in R (version 4.0) and PLINK (version 1.9).

## 3. Results

The current study included 346,352 unrelated individuals of European descent (Figure 1). The median age of the included participants was 58 years (interquartile range [IQR]: 51–63), and 53.34% were female. The characteristics of the study population are summarized in Table 1. A total of 39,963 IBS patients were identified. Compared with individuals without IBS, IBS patients were much younger and were more likely to be female.

### 3.1. Phenome-Wide Association Analyses

PRS-predicted IBS in the target sample, as calculated based on the GWAS statistics obtained from the base sample, was significantly associated with phenotypic IBS. The risk of incident IBS would increase 10% with one standard deviation (SD) increase in the PRS of IBS (odds ratio (OR) 1.10, 95% confidence interval (CI), 1.08–1.11, *p* = 9.22 × 10^−34^). When the PRS of IBS was further categorized into three groups according to the tertiles of PRS, the risk of developing IBS in the high-genetic-risk group increased by 23% (OR = 1.23, 95% CI 1.18–1.27, *p* = 5.68 × 10^−28^) compared with the low genetic risk group. Genetically predicted IBS was not correlated with common confounders including age, sex, body mass index (BMI), smoking, alcohol consumption, and genetic PC (Table S5).

A total of 665 disease groups were included in the PheWAS analyses. We found that one SD increase in the PRS of IBS was significantly associated with the risk of diverticular diseases of the intestine (OR 1.04, 95% CI, 1.02–1.05, *p* = 1.12 × 10^−7^), depression (OR 1.05, 95% CI, 1.03–1.07, *p* = 7.54 × 10^−6^), GERD (OR 1.04, 95% CI, 1.02–1.05, *p* = 1.08 × 10^−5^), dyspepsia (OR 1.07, 95% CI, 1.04–1.10, *p* = 9.83 × 10^−7^), and diaphragmatic hernia (OR 1.04, 95% CI, 1.02–1.05, *p* = 7.89 × 10^−6^) (Table 2).

### 3.2. Mendelian Randomization Analyses

**Figure 2:**
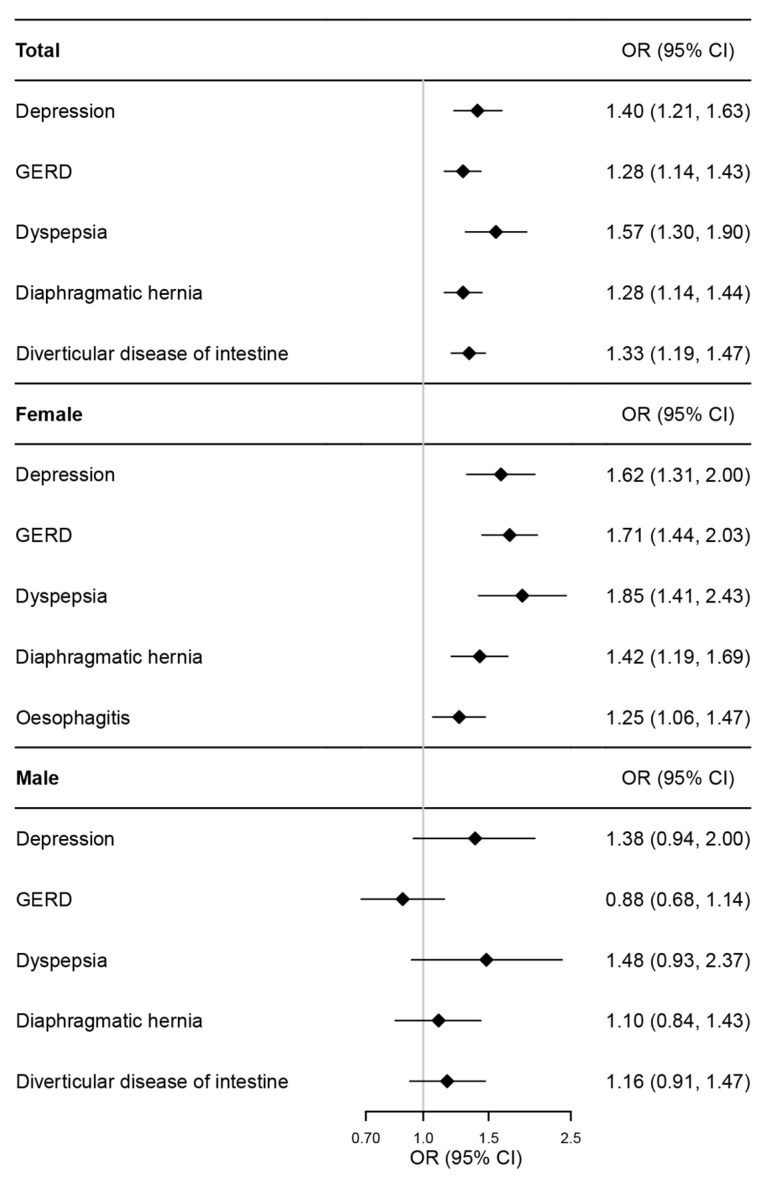
Forest plot of individual-level MR estimates for association between IBS and the outcomes identified in PheWAS in total and sex-stratified population. IBS—irritable bowel syndrome; PheWAS—phenome-wide association study; MR—Mendelian randomization; OR—odds ratio; CI—confidence interval; GERD—gastro-esophageal reflux disease.

Individual-level MR was performed to infer causal relationships among all outcomes identified in the initial PheWAS analysis. As shown in Figure 2 and Table S6, per doubling the odds of IBS was significantly correlated with increased risks of all outcomes identified in PheWAS. For example, per doubling the odds of IBS was associated with depression (OR 1.40, 95% CI, 1.21–1.63, *p* = 1.10 × 10^−5^), GERD (OR 1.28, 95% CI, 1.14–1.43, *p* = 2.55 × 10^−5^), dyspepsia (OR 1.57, 95% CI, 1.30–1.90, *p* = 3.23 × 10^−6^), diaphragmatic hernia (OR 1.28, 95% CI, 1.14–1.44, *p* = 2.11 × 10^−5^), and diverticular disease of the intestine (OR 1.33, 95% CI, 1.19–1.47, *p* = 2.03 × 10^−7^).

**Figure 3:**
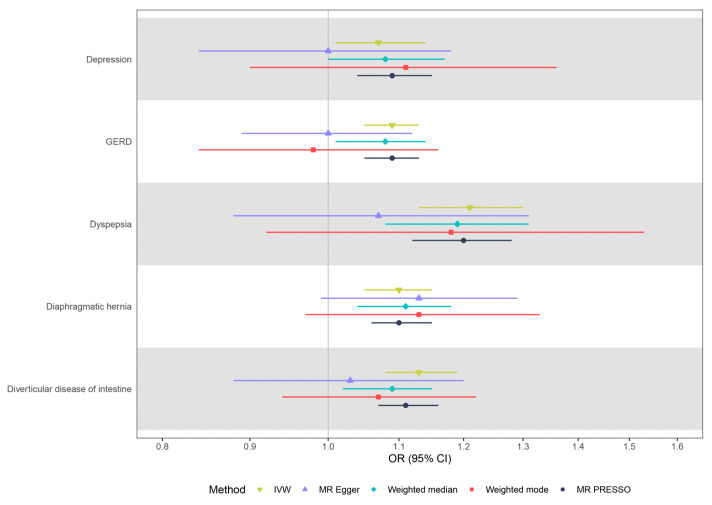
Summary-level MR estimates from each method for the assessment of the causal effects of IBS on five disease outcomes. IBS—irritable bowel syndrome; MR—Mendelian randomization; OR—odds ratio; CI—confidence interval; GERD—gastro-esophageal reflux disease; IVW—inverse-variance.

As shown in Figure 3 and Table S7, the results from summary-level MR were consistent with the results from individual-level MR. Per doubling the odds of IBS was associated with depression (OR: 1.07, 95% CI: 1.01–1.14, *p* = 0.02), diverticular diseases of the intestine (OR: 1.13, 95% CI: 1.08–1.19, *p* = 3.00 × 10^−6^), GERD (OR: 1.09, 95% CI: 1.05–1.13, *p* = 3.72 × 10^−5^), dyspepsia (OR: 1.21, 95% CI: 1.13–1.30, *p* = 9.28 × 10^−8^), and diaphragmatic hernia (OR: 1.10, 95% CI: 1.05–1.15, *p* = 2.75e × 10^−5^). MR-Egger analysis indicated that there was no unbalanced pleiotropy (*P* _Egger intercept_ > 0.05), and the correlations between IBS and health outcomes were consistent with the above-mentioned MR results, but with wider confidence intervals.

### 3.3. Sex-Stratified PheWAS and MR Analyses

The sex-stratified PheWAS analysis showed that, in women, consistent with the general population, per one SD increase in PRS of IBS was associated with depression, GERD, dyspepsia, and diaphragmatic hernia (Table 2) but not with diverticular diseases of the intestine. Additionally, a new association between IBS and esophagitis was identified in women (Table 2). The causality of these associations was confirmed by a one-sample MR in female participants (Figure 2 and Table S6). However, no significant association pairs were identified in men.

### 3.4. Sensitivity Analysis

Results from the weighted median, weighted mode, and MR PRESSO methods supported the causal associations between IBS and these five disease outcomes, further supporting the robustness of the primary analysis findings. Potential heterogeneity among the genetic instruments was observed in the correlation between IBS and diverticular disease (Figure 3, Table S7). The MR Steiger directionality test provided evidence to support the correct directions from IBS to these five disease outcomes (Figure 3, Table S7). Visual inspection of the scatter and leave-one-out plots did not suggest biased estimates or pleiotropy among patients with IBS and the five identified outcomes (Figures S1 and S2). After manual exclusion of three potential pleiotropic genetic variants simultaneously associated with IBS and diverticular disease (Table S8), genetically predicted IBS was significantly associated with diverticular disease (OR: 1.008, 95% CI: 1.005–1.012, *p* = 1.86 × 10^−7^), although the possible heterogeneity still remained.

The causal associations between IBS and the five identified outcomes were replicated in additional, publicly available GWAS statistics of outcomes (Table S9). However, potential pleiotropic effects were observed in the causal association between IBS and depression, as well as diverticular disease (*P*_Egger intercept_ < 0.05) (Table S9).

Subsequently, a reversed MR analysis was performed, and putative bidirectional causal associations between depression, GERD, diaphragmatic hernia, diverticular disease, and IBS were identified. Cochran’s Q test indicated significant heterogeneity among genetic variants in the causal associations between depression, diaphragmatic hernia, diverticular disease, and IBS (Table S10).

## 4. Discussion

In this comprehensive phenome-wide MR study, we found that genetically predicted IBS was significantly associated with depression, GERD, dyspepsia, diaphragmatic hernia, and diverticular disease of the intestine on a phenome-wide scale, with robust evidence for the causal relationship based on MR analyses using different approaches accounting for bias of weak instruments, heterogeneity, and directional pleiotropy. We also noticed significant sex discrepancy in the PheWAS-MR analyses for IBS. The above-mentioned causal associations, as well as an additional association between IBS and esophagitis, were identified in women only, but not in men.

In the present study, we observed a causal relationship between IBS and digestive disorders. IBS was causally associated with dyspepsia but not vice versa, as supported by the IVW, weighted median, and MR PRESSO methods without potential pleiotropy, as well as replicated causality using publicly available GWAS data. This finding was consistent with that of previous observational studies in which IBS was identified as one of the risk factors for dyspepsia [29], and Bulgarian adults with IBS were reported to have a significantly higher prevalence of functional dyspepsia [30]. Sex differences were also reported in an observational study showing that female sex is correlated with a higher risk of dyspepsia in patients with IBS [31].

We also found bidirectional causal associations between IBS and diaphragmatic hernia and intestinal diverticular disease. However, significant heterogeneity among the instrumental variables was observed between these two diseases and IBS, indicating possible pleiotropy. IBS and hernia have similar symptoms, such as abdominal pain, constipation, and diarrhea, but few studies have focused on the association between these two diseases. One study focused on 558 patients with small bowel hernia and observed that IBS was more common in these patients than in those without enterocele [32]. Although the pathogenesis of IBS and diverticular disease remains incompletely understood, substantial overlap or coexistence of these two diseases has been observed. IBS was significantly more prevalent in patients with recurrent diverticulitis after sigmoidectomy. One previous study showed that patients over the age of 60 with symptomatic uncomplicated diverticular disease were associated with increased risks of abdominal pain and IBS symptoms [33].

Bidirectional causal associations between IBS and GERD were identified without potential heterogeneity or pleiotropy. GERD and IBS are common gastrointestinal disorders and are often concurrent in the same patient, and a significant association between IBS and GERD was found [34]. IBS was also reported to be associated with a poor response to proton pump inhibitor therapy in GERD patients [35]. Genetic similarities between IBS and GERD have been demonstrated; for example, polymorphisms in the GNB3 gene have been reported to be associated with both IBS [36] and GERD [37]. In addition, similarities in gastrointestinal sensory–motor abnormalities between IBS and GERD may be another reason for the high concurrence and bidirectional causality of these two diseases [37].

Although physiological and etiologic associations between IBS and these digestive disorders have not been fully investigated, some common risk factors, including psychological factors, dietary factors, gut microbiota, infection, and involvement of the immune system, were found to be involved in both IBS and these digestive disorders. These stimuli may lead to abnormal immune function of the mucosa, impaired intestinal mucosal barrier function, and increased intestinal sensitivity and permeability, which further increases the severity of these digestive diseases.

Our study provides evidence to support previous findings that bidirectional causal associations exist between IBS and depression [9], although significant heterogeneity among genetic instruments was observed in the causation from depression to IBS. Psychological disorders have been demonstrated to be risk factors for gastrointestinal diseases. Compared with healthy controls, the occurrence of mental disorders is more common and severe in patients with IBS. The bidirectional association between these two diseases is consistent with the gut–brain axis theory that IBS may facilitate the presence of a series of psychological symptoms, further aggravating gastrointestinal tract symptoms [1]. Psychological symptoms may lead to changes in intestinal motility and secretion and cause visceral hypersensitivity and psychological symptoms initiated by dysfunction of the mucosal barrier; immune activation also increases susceptibility to IBS [38]. In turn, bacteria and their metabolic products in the gut may influence the vagus nerve, immune system, permeability of the blood–brain barrier, and neuroinflammation, further exacerbating psychological symptoms and behaviors [39]. Therefore, it is speculated that the existence of the gut–brain axis, microbiome dysfunction, genetic factors, and infections may be implicated in the pathophysiology of the interaction between IBS and depression.

Sex differences have been widely reported in gastrointestinal diseases. IBS is more common in women [40], and female sex is associated with a high risk of IBS incidence [41] and other gastrointestinal diseases [42]. At the genetic level, a previous GWAS of IBS identified a sex-specific variant, which only increased the risk of IBS in women, but not in men [16]. However, few studies have addressed sex differences in IBS causality across the phenome-wide scale. Our study highlighted the sex disparities in the associations between IBS and other health outcomes. In women, we found IBS was associated with depression, esophagitis, GERD, dyspepsia, and diaphragmatic hernia, whereas in men, none of the above-mentioned associations were observed. Further one-sample MR results identified a causal link between IBS and depression, esophagitis, GERD, dyspepsia, and diaphragmatic hernia in women. Patients with IBS have higher estradiol levels than healthy controls [43]; therefore, it is postulated that sex hormones, including estrogens and androgens, may play important regulatory roles in patients with IBS [40]. Sex hormones may influence the peripheral and central regulatory functions of the gut–brain axis, leading to different visceral sensitivity, motility, intestinal barrier function, and immune activation of the intestinal mucosa [44], which may be the factors responsible for the sex differences in the association between IBS and its related health conditions. We also noticed the association between IBS and diverticular disease in women was no longer significant after the Bonferroni correction in PheWAS, which might be attributed to reduced statistical power caused by decreased sample size in subgroup analysis.

Our study has several strengths. First, we systematically explored the association between genetically predicted IBS and health outcomes on a phenome-wide scale. Compared to hypothesis-driven MR studies that consider only a single disease outcome, this is the first PheWAS-MR study to explore the causal effects of IBS across a broad-spectrum of diseases. Although randomized controlled trials remain the gold standard for testing causality, MR provides an alternative method to investigate causality in a time- and cost-saving manner. Second, it has been reported that sample overlap may lead to inflation of type 1 error when calculating PRS [17]. Therefore, we divided the 346,352 participants into two groups and calculated the effect sizes of instrumental variables in the base data and the PRS as well as PheWAS in the target data. However, in two-sample MR analysis, genetic instruments derived from GWAS analysis require a much larger sample size to achieve adequate statistical power. Therefore, we used publicly available GWAS summary data of IBS instead of GWAS summary statistics in the base data. Third, more IVs were selected as we relaxed the threshold of the *p* value when selecting IVs, which provide increased power and facilitate testing for horizontal pleiotropy. Fourth, we employed comprehensive and complementary sensitivity analysis strategies to overcome the infallible causality that results from pleiotropy. Finally, this is the first study to investigate sex differences in the causal association of IBS on a phenome-wide scale, and one new causality of IBS has been identified in females.

However, our study has some limitations. First, genetic instruments for IBS were derived from a meta-analysis that included U.K. Biobank participants; no available GWAS summary data excluding U.K. Biobank participants could be retrieved. There may be some sample overlap between exposure and outcomes; therefore, various sensitivity analyses were performed in our study. Second, summary-level MR of IBS stratified by sex was not performed because of the lack of available sex-stratified GWAS data. Third, although we excluded potential pleiotropic SNPs simultaneously correlated with IBS and diverticular disease, heterogeneity still exists between IBS and diverticular disease. How these heterogeneous SNPs influence causality needs to be explored further. Fourth, the potential molecular mechanisms implicated in causal association remain unclear, and some experimental methods are expected to be carried out in the future. Finally, our study was restricted to participants of European descent; therefore, the generalizability of the results to other ethnic groups should be investigated further.

In conclusion, this phenome-wide MR analysis provided putative evidence of the causal association between IBS and increased risks of depression, GERD, dyspepsia, diaphragmatic hernia, and diverticular disease of the intestine. New causal association between IBS and esophagitis together with putative causal association between IBS and depression, GERD, dyspepsia, and diaphragmatic hernia were found in women only, but not in men. These findings provide a rationale for further investigation of the molecular mechanisms that contribute to the causal associations between IBS and various health outcomes.

## Figures and Tables

**Table 1:** Baseline characteristics of the study cohort. *p* value less than 0.05 represents the significant differences among groups using chi-square test.

Baseline Characteristics	Total (*n* = 346,352)	IBS (*n* = 39,963)	No IBS (*n* = 306,389)	*p* Value
Age at recruitment (years), Mean (SD)	56.98 (7.88)	55.88 (7.73)	57.12 (7.89)	
Age at recruitment, *n* (%)				<0.001
<55 years	125,241 (36.16%)	16,489 (41.26%)	108,752 (35.49%)	
55–64 years	153,097 (44.20%)	17,695 (44.28%)	135,402 (44.19%)	
≥65 years	68,014 (19.64%)	5779 (14.46%)	62,235 (20.31%)	
Sex				<0.001
Male	161,621 (46.66%)	11,585 (28.99%)	156,353 (51.03%)	
Female	184,731 (53.34%)	28,378 (71.01%)	150,036 (48.97%)	
Townsend quintile				0.06
1	86,491 (24.97%)	10,163 (25.43%)	76,328 (24.91%)	
2	86,488 (24.97%)	9800 (24.52%)	76,688 (25.03%)	
3	86,477 (24.97%)	9979 (24.97%)	76,498 (24.97%)	
4	86,476 (24.97%)	9967 (24.94%)	76,509 (24.97%)	
Unknown	420 (0.12%)	54 (0.14%)	366 (0.12%)	
BMI, Mean (SD)	27.42 (4.75)	27.13 (5.02)	27.45 (4.72)	
BMI, *n* (%)				<0.001
Normal (<25 kg/m^2^)	113,826 (32.86%)	15,072 (37.71%)	98,754 (32.23%)	
Overweight (25–30 kg/m^2^)	147,662 (42.63%)	15,492 (38.77%)	132,170 (43.14%)	
Obese (≥30 kg/m^2^)	83,765 (24.18%)	9282 (23.23%)	74,483 (24.31%)	
Unknown	1099 (0.32%)	117 (0.29%)	982 (0.32%)	
Smoking status, *n* (%)				<0.001
Never	188,601 (54.45%)	22,398 (56.05%)	166,203 (54.25%)	
Previous	121,858 (35.18%)	14,098 (35.28%)	107,760 (35.17%)	
Current	34,709 (10.02%)	3374 (8.44%)	31,335 (10.23%)	
Unknown	1184 (0.34%)	93 (0.23%)	1091 (0.36%)	
Alcohol status, *n* (%)				<0.001
Never	10,517 (3.04%)	1286 (3.22%)	9231 (3.01%)	
Previous	11,844 (3.42%)	1664 (4.16%)	10,180 (3.32%)	
Current	323,685 (93.46%)	36,985 (92.55%)	286,700 (93.57%)	
Unknown	306 (0.09%)	28 (0.07%)	278 (0.09%)	

IBS—irritable bowel syndrome; PheWAS—phenome-wide association study; SD—standard deviation; OR—odds ratio; CI—confidence interval; BMI—body mass index.

**Table 2:** PheWAS results in the total population and in female and male participants.

Description	PheWAS			
	IBS (*n*)	No IBS (*n*)	OR (95% CI)	*p*
Total Population (*N* = 173,176)				
Depression	10,163	137,939	1.05 (1.03–1.07)	7.54 × 10^−6^
GERD	18,830	129,272	1.04 (1.02–1.05)	1.08 × 10^−5^
Dyspepsia	6153	141,949	1.07 (1.04–1.10)	9.83 × 10^−7^
Diaphragmatic hernia	18,516	129,586	1.04 (1.02–1.05)	7.89 × 10^−6^
Diverticular disease of intestine	22,706	125,396	1.04 (1.02–1.05)	1.12 × 10^−7^
Female (*N* = 92,172)				
Depression	6398	73,306	1.06 (1.03–1.09)	8.02 × 10^−6^
Esophagitis	2726	76,978	1.08 (1.04–1.12)	7.28 × 10^−5^
GERD	10,287	69,417	1.07 (1.05–1.09)	8.52 × 10^−10^
Dyspepsia	3799	75,905	1.08 (1.04–1.11)	5.59 × 10^−6^
Diaphragmatic hernia	10,278	69,426	1.04 (1.02–1.07)	7.06 × 10^−5^
Male (*N* = 81,004)				
Depression	3765	64,633	1.03 (1.00–1.07)	0.06
GERD	8543	59,855	0.99 (0.97–1.01)	0.46
Dyspepsia	2354	66,044	1.04 (1.00–1.08)	0.08
Diaphragmatic hernia	8238	60,160	1.01 (0.99–1.04)	0.32
Diverticular disease of intestine	10,882	57,516	1.02 (1.00–1.04)	0.13

PheWAS—phenome-wide association study; OR—odds ratio; CI—confidence interval; GERD—gastro-esophageal reflux disease.

## Data Availability

Data from U.K. Biobank could be applied from https://www.ukbiobank.ac.uk/ (The UK Biobank Resource used in the current study was under application 54803 (approved on 29 October 2019)). The publicly available summary statistics can be downloaded from cited reference. The MR analysis code can be found at https://mrcieu.github.io/TwoSampleMR/articles/index.html (accessed on 15 July 2022).

## Supplementary Materials

The following supporting information can be downloaded at: https://www.mdpi.com/article/10.3390/jcm12031106/s1, Figure S1: Scatter plots to visualize the causal effect between IBS and depression, GERD, diaphragmatic hernia, dyspepsia and diverticular disease; Figure S2: Leave-one-out sensitivity analysis of the causal associations between IBS and depression, GERD, diaphragmatic hernia, dyspepsia and diverticular disease; Table S1: Exclusion criteria of ICD-10, self-reported and operative codes in IBS cases definition; Table S2: The number of cases in each disease category; Table S3: SNP information of instrumental variables of IBS used in summary-level MR analysis; Table S4: Power to detect associations between IBS and disease outcomes on available numbers of cases, considering different effect sizes for expected causal effect by genetically predicted IBS; Table S5: The association between PRS of IBS and common confounding factors; Table S6: Individual-level MR results between IBS and identified outcomes in total and sex-stratified population; Table S7: Summary-level MR results between IBS and outcomes identified in PheWAS; Table S8: Identified pleiotropic SNPs of the association between genetically predicted IBS and disease outcomes with the significance threshold FDR = 0.05 in the PheWAS analysis; Table S9: Sensitivity analysis of MR results by using additional publicly available GWAS summary data; Table S10: Bidirectional MR results with 5 identified disease conditions as exposure and IBS as outcome; Supplementary Methods [9,17,18,45,46,47,48,49,50,51,52,53,54,55,56,57].

## References

[1] Ford A.C., Lacy B.E., Talley N.J. (2017). Irritable Bowel Syndrome. N. Engl. J. Med..

[2] Chey W.D., Kurlander J., Eswaran S. (2015). Irritable bowel syndrome: A clinical review. JAMA.

[3] Ford A.C., Sperber A.D., Corsetti M., Camilleri M. (2020). Irritable bowel syndrome. Lancet.

[4] Camilleri M. (2021). Diagnosis and Treatment of Irritable Bowel Syndrome: A Review. JAMA.

[5] McPherson Z.E., Sørensen H.T., Horváth-Puhó E., Agar A., Coroneo M.T., White A., Francis I.C., Pasquale L.R., Kang J.H., Pettersson S. (2021). Irritable bowel syndrome and risk of glaucoma: An analysis of two independent population-based cohort studies. United Eur. Gastroenterol. J..

[6] Lee S.Y., Hwang H.R., Yi Y.H., Kim J.M., Kim Y.J., Lee J.G., Cho Y.H., Tak Y.J., Lee S.H., Park E.J. (2021). Association between Irritable Bowel Syndrome and Risk of Osteoporosis in Korean Premenopausal Women. Med. Princ. Pract..

[7] Mertsalmi T.H., But A., Pekkonen E., Scheperjans F. (2021). Irritable Bowel Syndrome and Risk of Parkinson’s Disease in Finland: A Nationwide Registry-Based Cohort Study. J. Park. Dis..

[8] Zamani M., Alizadeh-Tabari S., Zamani V. (2019). Systematic review with meta-analysis: The prevalence of anxiety and depression in patients with irritable bowel syndrome. Aliment. Pharmacol. Ther..

[9] Eijsbouts C., Zheng T., Kennedy N.A., Bonfiglio F., Anderson C.A., Moutsianas L., Holliday J., Shi J., Shringarpure S., Voda A.I. (2021). Genome-wide analysis of 53,400 people with irritable bowel syndrome highlights shared genetic pathways with mood and anxiety disorders. Nat. Genet..

[10] Chen J., Chen X., Xie Y., Sun Y., Wang X., Hesketh T. (2021). Irritable bowel syndrome and migraine: Evidence from Mendelian randomization analysis in the UK Biobank. Expert Rev. Gastroenterol. Hepatol..

[11] Freuer D., Linseisen J., Meisinger C. (2022). Asthma and the risk of gastrointestinal disorders: A Mendelian randomization study. BMC Med..

[12] Sun Y., Chen X., Wang S., Deng M., Xie Y., Wang X., Chen J., Hesketh T. (2021). Gluten-free Diet Reduces the Risk of Irritable Bowel Syndrome: A Mendelian Randomization Analysis. Front. Genet..

[13] Bao W., Qi L., Bao Y., Wang S., Li W. (2022). Alleviating insomnia should decrease the risk of irritable bowel syndrome: Evidence from Mendelian randomization. Front. Pharmacol..

[14] Aziz I., Simrén M. (2021). The overlap between irritable bowel syndrome and organic gastrointestinal diseases. Lancet Gastroenterol. Hepatol..

[15] de Bortoli N., Tolone S., Frazzoni M., Martinucci I., Sgherri G., Albano E., Ceccarelli L., Stasi C., Bellini M., Savarino V. (2018). Gastroesophageal reflux disease, functional dyspepsia and irritable bowel syndrome: Common overlapping gastrointestinal disorders. Ann. Gastroenterol..

[16] Bonfiglio F., Zheng T., Garcia-Etxebarria K., Hadizadeh F., Bujanda L., Bresso F., Agreus L., Andreasson A., Dlugosz A., Lindberg G. (2018). Female-Specific Association Between Variants on Chromosome 9 and Self-Reported Diagnosis of Irritable Bowel Syndrome. Gastroenterology.

[17] Choi S.W., Mak T.S., O’Reilly P.F. (2020). Tutorial: A guide to performing polygenic risk score analyses. Nat. Protoc..

[18] Jiang L., Zheng Z., Fang H., Yang J. (2021). A generalized linear mixed model association tool for biobank-scale data. Nat. Genet..

[19] Butler R. (2007). The ICD-10 General Equivalence Mappings. Bridging the translation gap from ICD-9. J. AHIMA.

[20] Veturi Y., Lucas A., Bradford Y., Hui D., Dudek S., Theusch E., Verma A., Miller J.E., Kullo I., Hakonarson H. (2021). A unified framework identifies new links between plasma lipids and diseases from electronic medical records across large-scale cohorts. Nat. Genet..

[21] Verma A., Bradford Y., Dudek S., Lucas A.M., Verma S.S., Pendergrass S.A., Ritchie M.D. (2018). A simulation study investigating power estimates in phenome-wide association studies. BMC Bioinform..

[22] Burgess S., Thompson S.G. (2011). Avoiding bias from weak instruments in Mendelian randomization studies. Int. J. Epidemiol..

[23] Burgess S., Butterworth A., Thompson S.G. (2013). Mendelian randomization analysis with multiple genetic variants using summarized data. Genet. Epidemiol..

[24] Bowden J., Davey Smith G., Burgess S. (2015). Mendelian randomization with invalid instruments: Effect estimation and bias detection through Egger regression. Int. J. Epidemiol..

[25] Hartwig F.P., Smith G.D., Bowden J. (2017). Robust inference in summary data Mendelian randomization via the zero modal pleiotropy assumption. Int. J. Epidemiol..

[26] Verbanck M., Chen C.Y., Neale B., Do R. (2018). Detection of widespread horizontal pleiotropy in causal relationships inferred from Mendelian randomization between complex traits and diseases. Nat. Genet..

[27] Burgess S., Labrecque J.A. (2018). Mendelian randomization with a binary exposure variable: Interpretation and presentation of causal estimates. Eur. J. Epidemiol..

[28] Burgess S. (2014). Sample size and power calculations in Mendelian randomization with a single instrumental variable and a binary outcome. Int. J. Epidemiol..

[29] Nwokediuko S.C., Ijoma U., Obienu O. (2012). Functional dyspepsia: Subtypes, risk factors, and overlap with irritable bowel syndrome in a population of african patients. Gastroenterol. Res. Pract..

[30] Nakov R., Dimitrova-Yurukova D., Snegarova V., Uzunova M., Lyutakov I., Ivanova M., Madzharova K., Valkov H., Hristova R., Ivanov K. (2020). Prevalence of Irritable Bowel Syndrome, Functional Dyspepsia and their Overlap in Bulgaria: A Population-Based Study. J. Gastrointest. Liver Dis..

[31] Kim Y.A., Cho Y.J., Kwak S.G. (2020). The Association between Helicobacter pylori Infection and Irritable Bowel Syndrome: A Meta-Analysis. Int. J. Environ. Res. Public Health.

[32] Brochard C., Ropert A., Chambaz M., Gouriou C., Cardaillac C., Grainville T., Bouguen G., Siproudhis L. (2020). Chronic pelvic pain and rectal prolapse invite consideration of enterocele. Color. Dis..

[33] Järbrink-Sehgal M.E., Andreasson A., Talley N.J., Agréus L., Song J.Y., Schmidt P.T. (2016). Symptomatic Diverticulosis Is Characterized by Loose Stools. Clin. Gastroenterol. Hepatol..

[34] Cheung T.K., Lam K.F., Hu W.H., Lam C.L., Wong W.M., Hui W.M., Lai K.C., Lam S.K., Wong B.C. (2007). Positive association between gastro-oesophageal reflux disease and irritable bowel syndrome in a Chinese population. Aliment. Pharmacol. Ther..

[35] Zerbib F., Belhocine K., Simon M., Capdepont M., Mion F., des Varannes S.B., Galmiche J.P. (2012). Clinical, but not oesophageal pH-impedance, profiles predict response to proton pump inhibitors in gastro-oesophageal reflux disease. Gut.

[36] Lee H.J., Lee S.Y., Choi J.E., Kim J.H., Sung I.K., Park H.S., Jin C.J. (2010). G protein beta3 subunit, interleukin-10, and tumor necrosis factor-alpha gene polymorphisms in Koreans with irritable bowel syndrome. Neurogastroenterol. Motil..

[37] de Vries D.R., ter Linde J.J., van Herwaarden M.A., Smout A.J., Samsom M. (2009). Gastroesophageal reflux disease is associated with the C825T polymorphism in the G-protein beta3 subunit gene (GNB3). Am. J. Gastroenterol..

[38] Staudacher H.M., Mikocka-Walus A., Ford A.C. (2021). Common mental disorders in irritable bowel syndrome: Pathophysiology, management, and considerations for future randomised controlled trials. Lancet Gastroenterol. Hepatol..

[39] Rutsch A., Kantsjö J.B., Ronchi F. (2020). The Gut-Brain Axis: How Microbiota and Host Inflammasome Influence Brain Physiology and Pathology. Front. Immunol..

[40] So S.Y., Savidge T.C. (2021). Sex-Bias in Irritable Bowel Syndrome: Linking Steroids to the Gut-Brain Axis. Front. Endocrinol..

[41] Narayanan S.P., Anderson B., Bharucha A.E. (2021). Sex- and Gender-Related Differences in Common Functional Gastroenterologic Disorders. Mayo Clin. Proc..

[42] Kovács D.B., Szekely A., Hubai A.G., Palsson O. (2022). Prevalence, epidemiology and associated healthcare burden of Rome IV irritable bowel syndrome and functional dyspepsia in the adult population of Gibraltar. BMJ Open Gastroenterol..

[43] Weaver K.R., Boulineaux C.M., Robinson J.M., Butler K., Heitkemper M.M., Henderson W.A. (2021). Sex Hormones, BDNF, Leptin, and TGF-β1 in Females With IBS: A Pilot Investigation. Biol. Res. Nurs..

[44] Mulak A., Taché Y., Larauche M. (2014). Sex hormones in the modulation of irritable bowel syndrome. World J. Gastroenterol..

[45] Abecasis G.R., Auton A., Brooks L.D., DePristo M.A., Durbin R.M., Handsaker R.E., Kang H.M., Marth G.T., McVean G.A. (2012). An integrated map of genetic variation from 1,092 human genomes. Nature.

[46] Reed E., Nunez S., Kulp D., Qian J., Reilly M.P., Foulkes A.S. (2015). A guide to genome-wide association analysis and post-analytic interrogation. Stat. Med..

[47] Tang L., Li C., Chen W., Zeng Y., Yang H., Hu Y., Song H., Zeng X., Li Q., Fu P. (2022). Causal Association between Chronic Kidney Disease and Risk of 19 Site-Specific Cancers: A Mendelian Randomization Study. Cancer Epidemiol. Biomark. Prev..

[48] Barbara G., Grover M., Bercik P., Corsetti M., Ghoshal U.C., Ohman L., Rajilić-Stojanović M. (2019). Rome Foundation Working Team Report on Post-Infection Irritable Bowel Syndrome. Gastroenterology.

[49] Akhondi N., Memar Montazerin S., Soltani S., Saneei P., Hassanzadeh Keshteli A., Esmaillzadeh A., Adibi P. (2019). General and abdominal obesity in relation to the prevalence of irritable bowel syndrome. Neurogastroenterol. Motil..

[50] Nilsson D., Ohlsson B. (2021). Gastrointestinal Symptoms and Irritable Bowel Syndrome Are Associated with Female Sex and Smoking in the General Population and with Unemployment in Men. Front. Med..

[51] Cozma-Petruţ A., Loghin F., Miere D., Dumitraşcu D.L. (2017). Diet in irritable bowel syndrome: What to recommend, not what to forbid to patients. World J. Gastroenterol..

[52] Hong E.P., Park J.W. (2012). Sample size and statistical power calculation in genetic association studies. Genom. Inform..

[53] Howard D.M., Adams M.J., Clarke T.K., Hafferty J.D., Gibson J., Shirali M., Coleman J., Hagenaars S.P., Ward J., Wigmore E.M. (2019). Genome-wide meta-analysis of depression identifies 102 independent variants and highlights the importance of the prefrontal brain regions. Nat. Neurosci..

[54] An J., Gharahkhani P., Law M.H., Ong J.S., Han X., Olsen C.M., Neale R.E., Lai J., Vaughan T.L., Gockel I. (2019). Gastroesophageal reflux GWAS identifies risk loci that also associate with subsequent severe esophageal diseases. Nat. Commun..

[55] Garcia-Etxebarria K., Carbone F., Teder-Laving M., Pandit A., Holvoet L., Thijs V., Lemmens R., Bujanda L., Franke A., Zöllner S. (2022). A survey of functional dyspepsia in 361,360 individuals: Phenotypic and genetic cross-disease analyses. Neurogastroenterol. Motil..

[56] Fadista J., Skotte L., Karjalainen J., Abner E., Sørensen E., Ullum H., Werge T., Esko T., Milani L., iPSYCH Group (2022). Comprehensive genome-wide association study of different forms of hernia identifies more than 80 associated loci. Nat. Commun..

[57] Maguire L.H., Handelman S.K., Du X., Chen Y., Pers T.H., Speliotes E.K. (2018). Genome-wide association analyses identify 39 new susceptibility loci for diverticular disease. Nat. Genet..

