# Supplementary material for: The Causal Association of Irritable Bowel Syndrome with Multiple Disease Outcomes: A Phenome-Wide Mendelian Randomization Study
Source: J Clin Med. 2023 Jan 31;12(3):1106. doi: 10.3390/jcm12031106 (PMC9918111; doi:10.3390/jcm12031106)
Supplement: Supplementary file 1 [file jcm-12-01106-s001.zip › Supplementary figures and tables.pdf]

Table S1. Exclusion criteria of ICD-10, self-reported and operative codes in IBS cases definition. ICD-10, International Classification of Diseases version 10; IBS, irritable bowel syndrome.

ICD-10 codes	Descrption	Self-reported codes in UKB	Descrption	Operative procedures - OPCS4 codes	Descrption
K50	Crohn's disease [regional enteritis]	1462	crohns disease	G01	Excision of oesophagus and stomach
K500	Crohn's disease of small intestine	1463	ulcerative colitis	G02	Total excision of oesophagus
K501	Crohn's disease of large intestine	1459	colitis/not crohns or ulcerative colitis	G03	Partial excision of oesophagus
K508	Other Crohn's disease	1461	inflammatory bowel disease	G05	Bypass of oesophagus
K509	Crohn's disease, unspecified	1164	pancreatic disease	G06	Attention to connection of oesophagus
K51	Ulcerative colitis	1165	pancreatitis	G08	Artificial opening into oesophagus
K510	Ulcerative (chronic) enterocolitis	1456	malabsorption/coeliac disease	G27	Total excision of stomach
K511	Ulcerative (chronic) ileocolitis	1191	gastrointestinal bleeding	G28	Partial excision of stomach
K512	Ulcerative (chronic) proctitis	1509	gastroenteritis/dysentry	G31	Connection of stomach to duodenum
K513	Ulcerative (chronic) rectosigmoiditis	1600	bowel / intestinal perforation	G32	Connection of stomach to transposed jejunum
K514	Pseudopolyposis of colon	1601	bowel / intestinal infarction	G33	Other connection of stomach to jejunum
K515	Mucosal proctocolitis	1602	bowel / intestinal obstruction	G34	Artificial opening into stomach
K518	Other ulcerative colitis	1135	stomach disorder	G49	Excision of duodenum
K519	Ulcerative colitis, unspecified			G51	Bypass of duodenum

K52	Other non-infective gastro-enteritis and colitis	G58	Excision of jejunum
K520	Gastro-enteritis and colitis due to radiation	G60	Artificial opening into jejunum
K521	Toxic gastro-enteritis and colitis	G61	Bypass of jejunum
K522	Allergic and dietetic gastro-enteritis and colitis	G69	Excision of ileum
K523	Indeterminate colitis	G71	Bypass of ileum
K528	Other specified non-infective gastro-enteritis and colitis	G72	Other connection of ileum
K529	Non-infective gastro-enteritis and colitis, unspecified	G73	Attention to connection of ileum
K550	Acute vascular disorders of intestine	G74	Creation of artificial opening into ileum
K551	Chronic vascular disorders of intestine	G75	Attention to artificial opening into ileum
K627	Radiation proctitis	H04	Total excision of colon and rectum
K86	Other diseases of pancreas	H05	Total excision of colon
K860	Alcohol-induced chronic pancreatitis	H06	Extended excision of right hemicolon
K861	Other chronic pancreatitis	H07	Other excision of right hemicolon
K862	Cyst of pancreas	H08	Excision of transverse colon
K863	Pseudocyst of pancreas	H09	Excision of left hemicolon
K868	Other specified diseases of pancreas	H10	Excision of sigmoid colon
K869	Disease of pancreas, unspecified	H11	Other excision of colon

K90	Intestinal malabsorption	H13	Bypass of colon
K900	Coeliac disease	H14	Exteriorisation of caecum
K901	Tropical sprue	H29	Subtotal excision of colon
K903	Pancreatic steatorrhoea	H33	Excision of rectum
K904	Malabsorption due to intolerance, not elsewhere classified	H47	Excision of anus
K908	Other intestinal malabsorption	H57	Other operations on the anal sphincter to control continence
K909	Intestinal malabsorption, unspecified	H68	Diagnostic endoscopic examination of enteric pouch using colonoscope
C15	Malignant neoplasm of oesophagus	H69	Diagnostic endoscopic examination of enteric pouch using fibreoptic sigmoidoscope
C16	Malignant neoplasm of stomach	H70	Diagnostic endoscopic examination of enteric pouch using rigid sigmoidoscope
C17	Malignant neoplasm of small intestine		
C18	Malignant neoplasm of colon		
C19	Malignant neoplasm of rectosigmoid junction		
C20	Malignant neoplasm of rectum		
C21	Malignant neoplasm of anus and anal canal		
C22	Malignant neoplasm of liver and intrahepatic bile ducts		
C23	Malignant neoplasm of gallbladder		

C24	Malignant neoplasm of other and unspecified parts of biliary tract
C25	Malignant neoplasm of pancreas
C26	Malignant neoplasm of other and ill-defined digestive organs

Table S2. The number of cases in each disease category.

Disease Category	No. of Phecodes	Phecode Cases Distribution Minimum	Median	Mean	Maximum
Certain infectious and parasitic diseases	22	86	597	1497	6928
Congenital malformations, deformations and chromosomal abnormalities	16	86	113	150	379
Diseases of the blood and blood-forming organs and certain disorders involving the immune mechanism	19	13	443	1355	9465
Diseases of the circulatory system	59	83	1026	3280	51976
Diseases of the digestive system	63	86	1672	3834	22706
Diseases of the ear and mastoid process	16	111	491	680	3768
Diseases of the eye and adnexa	32	134	576	1802	13462
Diseases of the genitourinary system	62	93	881	1863	13197
Diseases of the musculoskeletal system and connective tissue	61	112	1035	2708	15690
Diseases of the nervous system	44	86	360	855	5977
Diseases of the respiratory system	46	96	535	1702	16080
Diseases of the skin and subcutaneous tissue	33	79	565	1085	5311
Endocrine, nutritional and metabolic diseases	31	90	733	2938	26121
Mental and behavioural disorders	29	65	318	1377	10163
Neoplasms	91	84	427	1064	10453
Pregnancy, childbirth and the puerperium	41	69	227	367	1969

Table S3. SNP information of instrumental variables of IBS used in summary-level MR analysis. SNP, single nucleotide polymorphism; EAF: effect allele frequency; SE: Standard Error.

SNP	Chromosome	Position	Effect allele	Other allele	EAF	Beta	SE	*P* value	N Case	N Control	F
rs117391602	8	143263875	A	G	0.0204	-0.1191	0.0269	9.53E-06	50019	358262	19.60284
rs11738752	5	124586111	A	C	0.7148	-0.0341	0.0077	9.32E-06	53400	433201	19.61224
rs10010358	4	5405805	A	C	0.2616	-0.0355	0.008	9.16E-06	53400	418587	19.69141
rs142888522	4	157994712	A	G	0.0112	-0.1556	0.0349	8.01E-06	50019	392122	19.8778
rs35356207	8	125129099	A	C	0.3191	0.033	0.0074	8.75E-06	53400	433201	19.88678
rs2055084	12	92428531	A	G	0.0631	0.0683	0.0153	8.43E-06	50019	355329	19.92776
rs2669290	8	5583585	A	G	0.9154	-0.0636	0.0142	8.17E-06	44437	300342	20.06031
rs74174359	19	5936112	A	G	0.0407	-0.0851	0.019	7.42E-06	50019	351140	20.06097
rs213368	3	60268028	T	C	0.1654	0.0413	0.0092	7.66E-06	53400	433201	20.15229
rs77327887	10	130223023	T	C	0.9635	-0.087	0.0193	6.65E-06	53400	411465	20.32001
rs80094675	2	98951593	A	G	0.0401	0.0789	0.0175	6.69E-06	53400	430957	20.32722
rs4559793	13	34720412	A	G	0.9516	-0.0785	0.0174	6.52E-06	50019	364170	20.35358
rs35094163	11	72465651	A	G	0.2323	0.037	0.0082	6.09E-06	53400	433201	20.3599
rs6799861	3	23257084	T	G	0.652	0.0325	0.0072	6.25E-06	53400	433201	20.37519
rs9874850	3	13919315	T	C	0.1447	-0.0447	0.0099	6.55E-06	53400	433201	20.38659
rs113742420	21	35046917	T	G	0.9856	-0.1445	0.032	6.54E-06	50019	362223	20.39087
rs79600573	14	80286922	T	C	0.0365	0.0877	0.0194	5.95E-06	50019	399016	20.43599
rs71615648	5	20895170	A	G	0.0312	-0.1185	0.0262	6.28E-06	40548	293220	20.45663
rs188488	5	36992990	A	G	0.5663	-0.0362	0.008	6.36E-06	44437	300342	20.47563
rs10231223	7	24469952	T	C	0.7341	-0.0353	0.0078	6.33E-06	53400	426079	20.48143

rs3134615	1	40362066	A	C	0.2486	-0.0358	0.0079	5.85E-06	53400	433201	20.53581
rs72676302	14	36912454	T	C	0.0493	-0.0767	0.0169	5.90E-06	50019	398719	20.59763
rs56336528	18	74185670	T	C	0.1004	-0.0563	0.0124	5.97E-06	50019	361926	20.61453
rs4697055	4	24267336	A	G	0.7904	-0.0383	0.0084	5.44E-06	53400	433201	20.78926
rs17210284	7	82516893	T	C	0.769	-0.037	0.0081	4.78E-06	53400	433201	20.86572
rs35418299	16	60721629	A	G	0.5058	0.0311	0.0068	4.76E-06	53400	433201	20.91717
rs2828078	21	24713845	A	G	0.1428	-0.0467	0.0102	4.45E-06	53400	422699	20.96203
rs13251888	8	82696068	A	G	0.0551	0.0747	0.0163	4.83E-06	50019	357276	21.00226
rs1968081	7	7610206	A	C	0.656	0.0335	0.0073	5.22E-06	53400	422699	21.0593
rs2731562	2	144383183	A	C	0.5117	-0.0317	0.0069	3.66E-06	53400	433201	21.1067
rs11533038	9	84856691	T	C	0.4113	0.0322	0.007	3.60E-06	53400	433201	21.16
rs2197542	2	119925686	T	G	0.1232	0.0484	0.0105	3.64E-06	53400	433201	21.24771
rs12358694	10	72990966	A	G	0.5337	-0.0328	0.0071	4.47E-06	53400	383378	21.3418
rs2123209	15	74145915	T	C	0.1094	0.0509	0.011	3.33E-06	53400	433201	21.41165
rs111491723	13	80110374	A	C	0.976	0.1153	0.0249	3.68E-06	50019	359979	21.44173
rs5009448	6	29940488	T	C	0.2773	-0.0366	0.0079	3.25E-06	53400	416724	21.46387
rs112215352	12	59795763	A	G	0.9542	-0.0794	0.0171	3.19E-06	53400	415577	21.56
rs72811605	5	166034826	A	G	0.9423	0.0773	0.0166	3.29E-06	44437	312628	21.68417
rs3096652	17	46762881	A	G	0.5029	-0.0326	0.007	3.03E-06	53400	422699	21.68898
rs150272003	12	124151841	T	G	0.9865	-0.1593	0.0342	3.28E-06	46130	343043	21.69598
rs1280622	3	136508008	A	C	0.5899	0.0322	0.0069	3.24E-06	53400	433201	21.77778
rs7751504	6	97425657	T	C	0.3822	-0.0328	0.007	2.96E-06	53400	433201	21.95592
rs940468	7	114008173	T	C	0.5752	0.0328	0.007	2.77E-06	53400	426079	21.95592
rs12727764	1	158982477	T	G	0.1602	-0.0437	0.0093	2.95E-06	53400	433201	22.07989

rs183066057	10	32553717	A	G	0.9531	-0.0945	0.0201	2.48E-06	40548	293220	22.10403
rs56884006	14	61620772	T	C	0.7783	-0.0433	0.0092	2.60E-06	50019	349640	22.15135
rs7326447	13	99408540	T	C	0.0261	0.106	0.0224	2.30E-06	53400	415577	22.39318
rs57810551	1	214526612	T	G	0.6391	-0.0336	0.0071	2.29E-06	53400	433201	22.39556
rs2728934	3	9463462	A	G	0.2621	-0.0374	0.0079	2.09E-06	53400	426079	22.41243
rs537793984	11	89795691	A	G	0.1522	0.0583	0.0123	2.02E-06	40548	293220	22.46606
rs10917847	1	163752376	A	G	0.4656	0.0332	0.007	1.88E-06	53400	422699	22.49469
rs56246023	3	159713073	T	G	0.1328	-0.0584	0.0123	2.02E-06	40548	293220	22.5432
rs17331469	9	120540904	T	G	0.1368	0.0473	0.0099	2.01E-06	53400	433201	22.82716
rs1631779	7	97946450	A	C	0.0788	-0.0611	0.0127	1.40E-06	53400	433201	23.14595
rs9313179	5	7189925	A	C	0.6256	-0.0342	0.0071	1.29E-06	53400	433201	23.20254
rs12974306	19	10830281	T	G	0.483	-0.0333	0.0069	1.35E-06	53400	433201	23.29112
rs6471719	8	59380723	T	C	0.7214	-0.0367	0.0076	1.43E-06	53400	433201	23.31873
rs13031167	2	212706980	T	G	0.0896	0.0826	0.0171	1.31E-06	44437	300342	23.33285
rs6887323	5	170848124	A	G	0.5418	-0.0334	0.0069	1.31E-06	53400	433201	23.43121
rs189713900	2	32329842	A	G	0.9844	-0.1528	0.0315	1.18E-06	50019	347693	23.5302
rs12692627	2	161873799	A	G	0.2838	-0.0373	0.0076	8.31E-07	53400	433201	24.08743
rs10268606	7	146689731	T	C	0.704	0.037	0.0075	8.36E-07	53400	433201	24.33778
rs13250534	8	40280428	T	G	0.1418	0.0523	0.0106	7.92E-07	53400	413630	24.34398
rs12464541	2	82353052	T	C	0.8001	-0.0421	0.0085	7.27E-07	53400	433201	24.53163
rs11656412	17	5155729	A	G	0.6096	0.0348	0.007	7.78E-07	53400	426079	24.7151
rs6696068	1	53740797	T	G	0.3891	-0.035	0.007	5.80E-07	53400	433201	25
rs20551	22	41548008	A	G	0.7149	-0.0375	0.0075	6.16E-07	53400	433201	25
rs10044618	5	87781168	T	C	0.4316	0.035	0.0069	4.12E-07	53400	433201	25.72989
rs2504511	1	176284750	A	G	0.4757	-0.0346	0.0068	3.83E-07	53400	433201	25.89014
rs5017929	8	94653283	A	G	0.7009	0.0387	0.0075	2.12E-07	53400	433201	26.6256

rs13321176	3	150944808	T	G	0.8298	0.0478	0.0092	1.80E-07	53400	433201	26.9948
rs2674053	2	59770395	T	C	0.8518	-0.0501	0.0096	2.04E-07	53400	433201	27.23535
rs8106322	19	32024230	A	G	0.646	-0.0394	0.0075	1.45E-07	53400	416343	27.59751
rs12549729	8	8523912	T	G	0.3898	0.0475	0.0086	2.88E-08	44437	310156	30.50635
rs9513519	13	99610146	A	G	0.6168	0.0388	0.007	3.09E-08	53400	433201	30.72327
rs2813573	13	53943333	T	C	0.4846	-0.0378	0.0068	3.31E-08	53400	433201	30.90052
rs7106434	11	112860579	T	C	0.4095	0.0384	0.0069	3.19E-08	53400	433201	30.97164
rs10156602	9	96345328	A	G	0.633	0.0417	0.0071	4.36E-09	53400	433201	34.49494
rs1248825	3	84993411	A	C	0.3253	0.0444	0.0073	1.20E-09	53400	430957	36.99306
rs1052486	6	31610686	A	G	0.525	-0.044	0.007	4.18E-10	53400	416724	39.5102

Table S4. Power to detect associations between IBS and disease outcomes on available numbers of cases, considering different effect sizes for expected causal effect by genetically predicted IBS. IBS, irritable bowel syndrome.

Phecodes	Description	N Cases	N control	Beta =0.1	Beta =0.2	Beta =0.3	Beta =0.4	Beta =0.5	Beta =0.6	Beta =0.7	Beta =0.8	Beta =0.9	Beta =1
A04	Other bacterial intestinal infections	1616	146486	0.04	0.07	0.12	0.17	0.25	0.34	0.43	0.54	0.63	0.73
A08	Viral and other specified intestinal infections	680	147422	0.04	0.05	0.07	0.1	0.13	0.17	0.21	0.27	0.32	0.38
A09	Diarrhoea and gastro-enteritis of presumed infectious origin	6928	141174	0.07	0.18	0.34	0.55	0.74	0.88	0.95	0.99	1	1
A40	Streptococcal septicaemia	283	147819	0.03	0.04	0.05	0.06	0.08	0.09	0.11	0.14	0.16	0.19
A41	Other septicaemia	5325	142777	0.07	0.15	0.28	0.45	0.63	0.79	0.89	0.96	0.98	1
A49	Bacterial infection of unspecified site	826	147276	0.04	0.06	0.08	0.11	0.15	0.2	0.25	0.31	0.38	0.45
A63	Other predominantly sexually transmitted diseases, not elsewhere classified	94	148008	0.03	0.03	0.04	0.04	0.05	0.06	0.06	0.07	0.08	0.09
B00	Herpesviral [herpes simplex] infections	217	147885	0.03	0.04	0.05	0.06	0.07	0.08	0.1	0.11	0.13	0.15
B02	Zoster [herpes zoster]	430	147672	0.03	0.05	0.06	0.08	0.1	0.12	0.15	0.18	0.22	0.26
B07	Viral warts	514	147588	0.03	0.05	0.06	0.08	0.11	0.14	0.17	0.21	0.26	0.3
B17	Other acute viral hepatitis	89	148013	0.03	0.03	0.04	0.04	0.05	0.06	0.06	0.07	0.08	0.09
B18	Chronic viral hepatitis	204	147898	0.03	0.04	0.05	0.06	0.07	0.08	0.09	0.11	0.13	0.15
B25	Cytomegaloviral disease	86	148016	0.03	0.03	0.04	0.04	0.05	0.05	0.06	0.07	0.08	0.09
B34	Viral infection of unspecified site	1211	146891	0.04	0.06	0.1	0.14	0.2	0.26	0.34	0.43	0.51	0.6
B35	Dermatophytosis	203	147899	0.03	0.04	0.05	0.06	0.07	0.08	0.09	0.11	0.13	0.15
B37	Candidiasis	2346	145756	0.05	0.09	0.15	0.23	0.34	0.45	0.58	0.69	0.79	0.87
B44	Aspergillosis	136	147966	0.03	0.04	0.04	0.05	0.06	0.07	0.08	0.09	0.1	0.11

B95	Streptococcus and staphylococcus as the cause of diseases classified to other chapters	2974	145128	0.05	0.1	0.18	0.28	0.41	0.55	0.68	0.79	0.88	0.93
B96	Other bacterial agents as the cause of diseases classified to other chapters	6341	141761	0.07	0.17	0.32	0.51	0.7	0.85	0.94	0.98	0.99	1
B97	Viral agents as the cause of diseases classified to other chapters	1257	146845	0.04	0.07	0.1	0.15	0.2	0.27	0.35	0.44	0.53	0.62
B98	Other specified infectious agents as the cause of diseases classified to other chapters	965	147137	0.04	0.06	0.09	0.12	0.17	0.22	0.28	0.35	0.43	0.51
B99	Other and unspecified infectious diseases	199	147903	0.03	0.04	0.05	0.05	0.07	0.08	0.09	0.11	0.13	0.15
C01	Malignant neoplasm of base of tongue	84	148018	0.03	0.03	0.04	0.04	0.05	0.05	0.06	0.07	0.08	0.08
C02	Malignant neoplasm of other and unspecified parts of tongue	154	147948	0.03	0.04	0.04	0.05	0.06	0.07	0.08	0.09	0.11	0.12
C09	Malignant neoplasm of tonsil	159	147943	0.03	0.04	0.04	0.05	0.06	0.07	0.08	0.09	0.11	0.12
C15	Malignant neoplasm of oesophagus	676	147426	0.04	0.05	0.07	0.1	0.13	0.17	0.21	0.26	0.32	0.38
C16	Malignant neoplasm of stomach	485	147617	0.03	0.05	0.06	0.08	0.1	0.13	0.16	0.2	0.24	0.29
C17	Malignant neoplasm of small intestine	162	147940	0.03	0.04	0.04	0.05	0.06	0.07	0.08	0.1	0.11	0.13
C18	Malignant neoplasm of colon	2383	145719	0.05	0.09	0.15	0.24	0.34	0.46	0.58	0.7	0.8	0.87
C19	Malignant neoplasm of rectosigmoid junction	599	147503	0.04	0.05	0.07	0.09	0.12	0.15	0.19	0.24	0.29	0.35
C20	Malignant neoplasm of rectum	1072	147030	0.04	0.06	0.09	0.13	0.18	0.24	0.31	0.39	0.47	0.55
C21	Malignant neoplasm of anus and anal canal	149	147953	0.03	0.04	0.04	0.05	0.06	0.07	0.08	0.09	0.1	0.12
C22	Malignant neoplasm of liver and intrahepatic bile ducts	435	147667	0.03	0.05	0.06	0.08	0.1	0.12	0.15	0.19	0.22	0.27
C24	Malignant neoplasm of other and unspecified parts of biliary tract	129	147973	0.03	0.03	0.04	0.05	0.06	0.06	0.07	0.08	0.1	0.11
C25	Malignant neoplasm of pancreas	666	147436	0.04	0.05	0.07	0.1	0.13	0.17	0.21	0.26	0.32	0.38

C26	Malignant neoplasm of other and ill-defined digestive organs	240	147862	0.03	0.04	0.05	0.06	0.07	0.09	0.1	0.12	0.14	0.17
C32	Malignant neoplasm of larynx	181	147921	0.03	0.04	0.04	0.05	0.06	0.07	0.09	0.1	0.12	0.14
C34	Malignant neoplasm of bronchus and lung	1988	146114	0.05	0.08	0.13	0.2	0.29	0.4	0.51	0.62	0.72	0.81
C43	Malignant melanoma of skin	1811	146291	0.05	0.08	0.13	0.19	0.27	0.37	0.47	0.58	0.68	0.77
C44	Other malignant neoplasms of skin	9069	139033	0.09	0.22	0.43	0.66	0.84	0.94	0.99	1	1	1
C45	Mesothelioma	221	147881	0.03	0.04	0.05	0.06	0.07	0.08	0.1	0.12	0.13	0.16
C48	Malignant neoplasm of retroperitoneum and peritoneum	154	147948	0.03	0.04	0.04	0.05	0.06	0.07	0.08	0.09	0.11	0.12
C49	Malignant neoplasm of other connective and soft tissue	222	147880	0.03	0.04	0.05	0.06	0.07	0.08	0.1	0.12	0.14	0.16
C50	Malignant neoplasm of breast	6530	141572	0.07	0.17	0.33	0.53	0.72	0.86	0.94	0.98	1	1
C51	Malignant neoplasm of vulva	88	148014	0.03	0.03	0.04	0.04	0.05	0.05	0.06	0.07	0.08	0.09
C53	Malignant neoplasm of cervix uteri	369	147733	0.03	0.04	0.06	0.07	0.09	0.11	0.14	0.16	0.2	0.23
C54	Malignant neoplasm of corpus uteri	888	147214	0.04	0.06	0.08	0.12	0.16	0.21	0.26	0.33	0.4	0.48
C55	Malignant neoplasm of uterus, part unspecified	103	147999	0.03	0.03	0.04	0.04	0.05	0.06	0.07	0.07	0.08	0.1
C56	Malignant neoplasm of ovary	697	147405	0.04	0.05	0.07	0.1	0.13	0.17	0.22	0.27	0.33	0.39
C57	Malignant neoplasm of other and unspecified female genital organs	106	147996	0.03	0.03	0.04	0.04	0.05	0.06	0.07	0.08	0.09	0.1
C61	Malignant neoplasm of prostate	5033	143069	0.07	0.14	0.27	0.43	0.61	0.76	0.88	0.95	0.98	0.99
C62	Malignant neoplasm of testis	278	147824	0.03	0.04	0.05	0.06	0.08	0.09	0.11	0.13	0.16	0.19
C64	Malignant neoplasm of kidney, except renal pelvis	898	147204	0.04	0.06	0.08	0.12	0.16	0.21	0.27	0.33	0.41	0.48
C66	Malignant neoplasm of ureter	94	148008	0.03	0.03	0.04	0.04	0.05	0.06	0.06	0.07	0.08	0.09
C67	Malignant neoplasm of bladder	1629	146473	0.04	0.07	0.12	0.18	0.25	0.34	0.44	0.54	0.64	0.73
C69	Malignant neoplasm of eye and adnexa	121	147981	0.03	0.03	0.04	0.05	0.05	0.06	0.07	0.08	0.09	0.1

C71	Malignant neoplasm of brain	427	147675	0.03	0.04	0.06	0.08	0.1	0.12	0.15	0.18	0.22	0.26
C73	Malignant neoplasm of thyroid gland	283	147819	0.03	0.04	0.05	0.06	0.08	0.09	0.11	0.14	0.16	0.19
C76	Malignant neoplasm of other and ill-defined sites	107	147995	0.03	0.03	0.04	0.05	0.05	0.06	0.07	0.08	0.09	0.1
C77	Secondary and unspecified malignant neoplasm of lymph nodes	4315	143787	0.06	0.13	0.24	0.38	0.54	0.7	0.83	0.91	0.96	0.99
C78	Secondary malignant neoplasm of respiratory and digestive organs	4469	143633	0.06	0.13	0.24	0.39	0.56	0.72	0.84	0.92	0.97	0.99
C79	Secondary malignant neoplasm of other sites	3768	144334	0.06	0.12	0.21	0.34	0.49	0.64	0.78	0.87	0.94	0.97
C80	Malignant neoplasm without specification of site	1368	146734	0.04	0.07	0.11	0.15	0.22	0.29	0.38	0.47	0.56	0.65
C81	Hodgkin's disease	210	147892	0.03	0.04	0.05	0.06	0.07	0.08	0.09	0.11	0.13	0.15
C82	Follicular [nodular] non-Hodgkin's lymphoma	350	147752	0.03	0.04	0.05	0.07	0.09	0.11	0.13	0.16	0.19	0.22
C83	Diffuse non-Hodgkin's lymphoma	679	147423	0.04	0.05	0.07	0.1	0.13	0.17	0.21	0.26	0.32	0.38
C84	Peripheral and cutaneous T-cell lymphomas	100	148002	0.03	0.03	0.04	0.04	0.05	0.06	0.07	0.07	0.08	0.09
C85	Other and unspecified types of non-Hodgkin's lymphoma	835	147267	0.04	0.06	0.08	0.11	0.15	0.2	0.25	0.31	0.38	0.45
C88	Malignant immunoproliferative diseases	94	148008	0.03	0.03	0.04	0.04	0.05	0.06	0.06	0.07	0.08	0.09
C90	Multiple myeloma and malignant plasma cell neoplasms	522	147580	0.03	0.05	0.06	0.08	0.11	0.14	0.17	0.21	0.26	0.31
C91	Lymphoid leukaemia	481	147621	0.03	0.05	0.06	0.08	0.1	0.13	0.16	0.2	0.24	0.29
C92	Myeloid leukaemia	399	147703	0.03	0.04	0.06	0.07	0.09	0.12	0.14	0.17	0.21	0.25
C97	Malignant neoplasms of independent (primary) multiple sites	528	147574	0.03	0.05	0.06	0.08	0.11	0.14	0.18	0.22	0.26	0.31
D03	Melanoma in situ	605	147497	0.04	0.05	0.07	0.09	0.12	0.15	0.19	0.24	0.29	0.35
D04	Carcinoma in situ of skin	667	147435	0.04	0.05	0.07	0.1	0.13	0.17	0.21	0.26	0.32	0.38

D05	Carcinoma in situ of breast	1323	146779	0.04	0.07	0.1	0.15	0.21	0.28	0.37	0.46	0.55	0.64
D06	Carcinoma in situ of cervix uteri	1535	146567	0.04	0.07	0.11	0.17	0.24	0.32	0.42	0.51	0.61	0.7
D07	Carcinoma in situ of other and unspecified genital organs	323	147779	0.03	0.04	0.05	0.07	0.08	0.1	0.12	0.15	0.18	0.21
D09	Carcinoma in situ of other and unspecified sites	333	147769	0.03	0.04	0.05	0.07	0.08	0.1	0.13	0.15	0.18	0.21
D10	Benign neoplasm of mouth and pharynx	1022	147080	0.04	0.06	0.09	0.13	0.17	0.23	0.3	0.37	0.45	0.53
D11	Benign neoplasm of major salivary glands	290	147812	0.03	0.04	0.05	0.06	0.08	0.1	0.12	0.14	0.16	0.19
D12	Benign neoplasm of colon, rectum, anus and anal canal	10453	137649	0.09	0.24	0.47	0.71	0.88	0.97	0.99	1	1	1
D13	Benign neoplasm of other and ill-defined parts of digestive system	777	147325	0.04	0.05	0.08	0.11	0.14	0.19	0.24	0.3	0.36	0.43
D14	Benign neoplasm of middle ear and respiratory system	158	147944	0.03	0.04	0.04	0.05	0.06	0.07	0.08	0.09	0.11	0.12
D16	Benign neoplasm of bone and articular cartilage	169	147933	0.03	0.04	0.04	0.05	0.06	0.07	0.08	0.1	0.11	0.13
D17	Benign lipomatous neoplasm	3561	144541	0.06	0.11	0.2	0.33	0.47	0.62	0.75	0.86	0.92	0.97
D18	Haemangioma and lymphangioma, any site	1022	147080	0.04	0.06	0.09	0.13	0.17	0.23	0.3	0.37	0.45	0.53
D21	Other benign neoplasms of connective and other soft tissue	354	147748	0.03	0.04	0.05	0.07	0.09	0.11	0.13	0.16	0.19	0.22
D22	Melanocytic naevi	2542	145560	0.05	0.09	0.16	0.25	0.36	0.48	0.61	0.73	0.82	0.89
D23	Other benign neoplasms of skin	2157	145945	0.05	0.09	0.14	0.22	0.31	0.43	0.54	0.66	0.76	0.84
D24	Benign neoplasm of breast	737	147365	0.04	0.05	0.07	0.1	0.14	0.18	0.23	0.28	0.34	0.41
D25	Leiomyoma of uterus	4826	143276	0.06	0.14	0.26	0.42	0.59	0.75	0.86	0.94	0.98	0.99
D26	Other benign neoplasms of uterus	188	147914	0.03	0.04	0.04	0.05	0.06	0.08	0.09	0.1	0.12	0.14
D27	Benign neoplasm of ovary	800	147302	0.04	0.05	0.08	0.11	0.15	0.19	0.24	0.3	0.37	0.44
D28	Benign neoplasm of other and unspecified female genital organs	118	147984	0.03	0.03	0.04	0.05	0.05	0.06	0.07	0.08	0.09	0.1

D29	Benign neoplasm of male genital organs	93	148009	0.03	0.03	0.04	0.04	0.05	0.06	0.06	0.07	0.08	0.09
D30	Benign neoplasm of urinary organs	135	147967	0.03	0.04	0.04	0.05	0.06	0.07	0.08	0.09	0.1	0.11
D31	Benign neoplasm of eye and adnexa	112	147990	0.03	0.03	0.04	0.05	0.05	0.06	0.07	0.08	0.09	0.1
D32	Benign neoplasm of meninges	452	147650	0.03	0.05	0.06	0.08	0.1	0.13	0.16	0.19	0.23	0.27
D33	Benign neoplasm of brain and other parts of central nervous system	248	147854	0.03	0.04	0.05	0.06	0.07	0.09	0.1	0.12	0.15	0.17
D34	Benign neoplasm of thyroid gland	148	147954	0.03	0.04	0.04	0.05	0.06	0.07	0.08	0.09	0.1	0.12
D35	Benign neoplasm of other and unspecified endocrine glands	744	147358	0.04	0.05	0.08	0.1	0.14	0.18	0.23	0.29	0.35	0.41
D36	Benign neoplasm of other and unspecified sites	393	147709	0.03	0.04	0.06	0.07	0.09	0.12	0.14	0.17	0.21	0.24
D37	Neoplasm of uncertain or unknown behaviour of oral cavity and digestive organs	826	147276	0.04	0.06	0.08	0.11	0.15	0.2	0.25	0.31	0.38	0.45
D38	Neoplasm of uncertain or unknown behaviour of middle ear and respiratory and intrathoracic organs	130	147972	0.03	0.03	0.04	0.05	0.06	0.06	0.07	0.08	0.1	0.11
D39	Neoplasm of uncertain or unknown behaviour of female genital organs	192	147910	0.03	0.04	0.05	0.05	0.06	0.08	0.09	0.11	0.12	0.14
D41	Neoplasm of uncertain or unknown behaviour of urinary organs	535	147567	0.03	0.05	0.06	0.09	0.11	0.14	0.18	0.22	0.26	0.32
D43	Neoplasm of uncertain or unknown behaviour of brain and central nervous system	179	147923	0.03	0.04	0.04	0.05	0.06	0.07	0.09	0.1	0.12	0.13
D44	Neoplasm of uncertain or unknown behaviour of endocrine glands	143	147959	0.03	0.04	0.04	0.05	0.06	0.07	0.08	0.09	0.1	0.12
D45	Polycythaemia vera	237	147865	0.03	0.04	0.05	0.06	0.07	0.09	0.1	0.12	0.14	0.16
D46	Myelodysplastic syndromes	283	147819	0.03	0.04	0.05	0.06	0.08	0.09	0.11	0.14	0.16	0.19

D47	Other neoplasms of uncertain or unknown behaviour of lymphoid, haematopoietic and related tissue	735	147367	0.04	0.05	0.07	0.1	0.14	0.18	0.23	0.28	0.34	0.41
D48	Neoplasm of uncertain or unknown behaviour of other and unspecified sites	448	147654	0.03	0.05	0.06	0.08	0.1	0.13	0.16	0.19	0.23	0.27
D50	Iron deficiency anaemia	7239	140863	0.08	0.18	0.36	0.57	0.76	0.89	0.96	0.99	1	1
D51	Vitamin B12 deficiency anaemia	653	147449	0.04	0.05	0.07	0.1	0.13	0.16	0.21	0.26	0.31	0.37
D52	Folate deficiency anaemia	263	147839	0.03	0.04	0.05	0.06	0.07	0.09	0.11	0.13	0.15	0.18
D56	Thalassaemia	41	148061	0.03	0.03	0.03	0.04	0.04	0.04	0.05	0.05	0.06	0.06
D57	Sickle-cell disorders	13	148089	0.03	0.03	0.03	0.03	0.03	0.03	0.04	0.04	0.04	0.04
D59	Acquired haemolytic anaemia	97	148005	0.03	0.03	0.04	0.04	0.05	0.06	0.06	0.07	0.08	0.09
D61	Other aplastic anaemias	458	147644	0.03	0.05	0.06	0.08	0.1	0.13	0.16	0.19	0.23	0.28
D62	Acute posthaemorrhagic anaemia	153	147949	0.03	0.04	0.04	0.05	0.06	0.07	0.08	0.09	0.11	0.12
D63	Anaemia in chronic diseases classified elsewhere	596	147506	0.04	0.05	0.07	0.09	0.12	0.15	0.19	0.24	0.29	0.34
D64	Other anaemias	9465	138637	0.09	0.23	0.44	0.67	0.85	0.95	0.99	1	1	1
D68	Other coagulation defects	654	147448	0.04	0.05	0.07	0.1	0.13	0.16	0.21	0.26	0.31	0.37
D69	Purpura and other haemorrhagic conditions	1650	146452	0.04	0.07	0.12	0.18	0.25	0.34	0.44	0.54	0.64	0.73
D70	Agranulocytosis	2425	145677	0.05	0.09	0.15	0.24	0.35	0.47	0.59	0.71	0.8	0.88
D72	Other disorders of white blood cells	365	147737	0.03	0.04	0.06	0.07	0.09	0.11	0.13	0.16	0.19	0.23
D73	Diseases of spleen	443	147659	0.03	0.05	0.06	0.08	0.1	0.12	0.15	0.19	0.23	0.27
D75	Other diseases of blood and blood-forming organs	493	147609	0.03	0.05	0.06	0.08	0.11	0.13	0.17	0.2	0.25	0.29
D80	Immunodeficiency with predominantly antibody defects	132	147970	0.03	0.03	0.04	0.05	0.06	0.06	0.07	0.09	0.1	0.11
D86	Sarcoidosis	368	147734	0.03	0.04	0.06	0.07	0.09	0.11	0.14	0.16	0.2	0.23

D89	Other disorders involving the immune mechanism, not elsewhere classified	235	147867	0.03	0.04	0.05	0.06	0.07	0.09	0.1	0.12	0.14	0.16
E03	Other hypothyroidism	9974	138128	0.09	0.23	0.46	0.7	0.87	0.96	0.99	1	1	1
E04	Other non-toxic goitre	1161	146941	0.04	0.06	0.1	0.14	0.19	0.26	0.33	0.41	0.5	0.58
E05	Thyrotoxicosis [hyperthyroidism]	1374	146728	0.04	0.07	0.11	0.16	0.22	0.29	0.38	0.47	0.57	0.66
E06	Thyroiditis	203	147899	0.03	0.04	0.05	0.06	0.07	0.08	0.09	0.11	0.13	0.15
E07	Other disorders of thyroid	336	147766	0.03	0.04	0.05	0.07	0.08	0.1	0.13	0.15	0.18	0.22
E10	Insulin-dependent diabetes mellitus	1626	146476	0.04	0.07	0.12	0.18	0.25	0.34	0.44	0.54	0.64	0.73
E11	Non-insulin-dependent diabetes mellitus	12929	135173	0.1	0.28	0.55	0.79	0.94	0.99	1	1	1	1
E13	Other specified diabetes mellitus	157	147945	0.03	0.04	0.04	0.05	0.06	0.07	0.08	0.09	0.11	0.12
E14	Unspecified diabetes mellitus	2095	146007	0.05	0.08	0.14	0.21	0.31	0.42	0.53	0.64	0.75	0.83
E16	Other disorders of pancreatic internal secretion	1090	147012	0.04	0.06	0.09	0.13	0.18	0.24	0.31	0.39	0.47	0.56
E21	Hyperparathyroidism and other disorders of parathyroid gland	733	147369	0.04	0.05	0.07	0.1	0.14	0.18	0.23	0.28	0.34	0.41
E22	Hyperfunction of pituitary gland	304	147798	0.03	0.04	0.05	0.07	0.08	0.1	0.12	0.14	0.17	0.2
E23	Hypofunction and other disorders of pituitary gland	279	147823	0.03	0.04	0.05	0.06	0.08	0.09	0.11	0.13	0.16	0.19
E27	Other disorders of adrenal gland	461	147641	0.03	0.05	0.06	0.08	0.1	0.13	0.16	0.19	0.23	0.28
E28	Ovarian dysfunction	148	147954	0.03	0.04	0.04	0.05	0.06	0.07	0.08	0.09	0.1	0.12
E29	Testicular dysfunction	119	147983	0.03	0.03	0.04	0.05	0.05	0.06	0.07	0.08	0.09	0.1
E34	Other endocrine disorders	98	148004	0.03	0.03	0.04	0.04	0.05	0.06	0.06	0.07	0.08	0.09
E46	Unspecified protein-energy malnutrition	186	147916	0.03	0.04	0.04	0.05	0.06	0.08	0.09	0.1	0.12	0.14
E53	Deficiency of other B group vitamins	1087	147015	0.04	0.06	0.09	0.13	0.18	0.24	0.31	0.39	0.47	0.56
E55	Vitamin D deficiency	1533	146569	0.04	0.07	0.11	0.17	0.24	0.32	0.42	0.51	0.61	0.7
E61	Deficiency of other nutrient elements	372	147730	0.03	0.04	0.06	0.07	0.09	0.11	0.14	0.17	0.2	0.23
E66	Obesity	12149	135953	0.1	0.27	0.53	0.77	0.92	0.98	1	1	1	1

E73	Lactose intolerance	90	148012	0.03	0.03	0.04	0.04	0.05	0.06	0.06	0.07	0.08	0.09
E78	Disorders of lipoprotein metabolism and other lipidaemias	26121	121981	0.15	0.47	0.8	0.96	1	1	1	1	1	1
E80	Disorders of porphyrin and bilirubin metabolism	379	147723	0.03	0.04	0.06	0.07	0.09	0.11	0.14	0.17	0.2	0.24
E83	Disorders of mineral metabolism	2901	145201	0.05	0.1	0.17	0.28	0.4	0.54	0.67	0.78	0.87	0.93
E85	Amyloidosis	180	147922	0.03	0.04	0.04	0.05	0.06	0.07	0.09	0.1	0.12	0.14
E86	Volume depletion	3628	144474	0.06	0.12	0.21	0.33	0.48	0.63	0.76	0.86	0.93	0.97
E87	Other disorders of fluid, electrolyte and acid-base balance	7859	140243	0.08	0.2	0.38	0.6	0.79	0.91	0.97	0.99	1	1
E88	Other metabolic disorders	448	147654	0.03	0.05	0.06	0.08	0.1	0.13	0.16	0.19	0.23	0.27
E89	Postprocedural endocrine and metabolic disorders, not elsewhere classified	1067	147035	0.04	0.06	0.09	0.13	0.18	0.24	0.31	0.38	0.47	0.55
F00	Dementia in Alzheimer's disease	833	147269	0.04	0.06	0.08	0.11	0.15	0.2	0.25	0.31	0.38	0.45
F01	Vascular dementia	626	147476	0.04	0.05	0.07	0.09	0.12	0.16	0.2	0.25	0.3	0.36
F02	Dementia in other diseases classified elsewhere	318	147784	0.03	0.04	0.05	0.07	0.08	0.1	0.12	0.15	0.18	0.21
F03	Unspecified dementia	1444	146658	0.04	0.07	0.11	0.16	0.23	0.31	0.4	0.49	0.59	0.68
F05	Delirium, not induced by alcohol and other psychoactive substances	2395	145707	0.05	0.09	0.15	0.24	0.34	0.46	0.59	0.7	0.8	0.87
F06	Other mental disorders due to brain damage and dysfunction and to physical disease	428	147674	0.03	0.04	0.06	0.08	0.1	0.12	0.15	0.18	0.22	0.26
F10	Mental and behavioural disorders due to use of alcohol	3692	144410	0.06	0.12	0.21	0.34	0.48	0.63	0.77	0.87	0.93	0.97
F11	Mental and behavioural disorders due to use of opioids	65	148037	0.03	0.03	0.04	0.04	0.04	0.05	0.05	0.06	0.07	0.07

F12	Mental and behavioural disorders due to use of cannabinoids	96	148006	0.03	0.03	0.04	0.04	0.05	0.06	0.06	0.07	0.08	0.09
F17	Mental and behavioural disorders due to use of tobacco	8225	139877	0.08	0.2	0.39	0.62	0.81	0.92	0.98	0.99	1	1
F20	Schizophrenia	320	147782	0.03	0.04	0.05	0.07	0.08	0.1	0.12	0.15	0.18	0.21
F22	Persistent delusional disorders	153	147949	0.03	0.04	0.04	0.05	0.06	0.07	0.08	0.09	0.11	0.12
F23	Acute and transient psychotic disorders	76	148026	0.03	0.03	0.04	0.04	0.05	0.05	0.06	0.07	0.07	0.08
F25	Schizoaffective disorders	81	148021	0.03	0.03	0.04	0.04	0.05	0.05	0.06	0.07	0.07	0.08
F29	Unspecified nonorganic psychosis	138	147964	0.03	0.04	0.04	0.05	0.06	0.07	0.08	0.09	0.1	0.11
F30	Manic episode	87	148015	0.03	0.03	0.04	0.04	0.05	0.05	0.06	0.07	0.08	0.09
F31	Bipolar affective disorder	604	147498	0.04	0.05	0.07	0.09	0.12	0.15	0.19	0.24	0.29	0.35
F32	Depressive episode	10163	137939	0.09	0.24	0.46	0.7	0.88	0.96	0.99	1	1	1
F33	Recurrent depressive disorder	400	147702	0.03	0.04	0.06	0.07	0.09	0.12	0.14	0.17	0.21	0.25
F40	Phobic anxiety disorders	745	147357	0.04	0.05	0.08	0.1	0.14	0.18	0.23	0.29	0.35	0.41
F41	Other anxiety disorders	7479	140623	0.08	0.19	0.37	0.58	0.77	0.9	0.97	0.99	1	1
F42	Obsessive-compulsive disorder	120	147982	0.03	0.03	0.04	0.05	0.05	0.06	0.07	0.08	0.09	0.1
F43	Reaction to severe stress, and adjustment disorders	411	147691	0.03	0.04	0.06	0.07	0.09	0.12	0.15	0.18	0.21	0.25
F44	Dissociative [conversion] disorders	98	148004	0.03	0.03	0.04	0.04	0.05	0.06	0.06	0.07	0.08	0.09
F45	Somatoform disorders	248	147854	0.03	0.04	0.05	0.06	0.07	0.09	0.1	0.12	0.15	0.17
F52	Sexual dysfunction, not caused by organic disorder or disease	255	147847	0.03	0.04	0.05	0.06	0.07	0.09	0.11	0.13	0.15	0.17
F60	Specific personality disorders	203	147899	0.03	0.04	0.05	0.06	0.07	0.08	0.09	0.11	0.13	0.15
F81	Specific developmental disorders of scholastic skills	107	147995	0.03	0.03	0.04	0.05	0.05	0.06	0.07	0.08	0.09	0.1
F99	Mental disorder, not otherwise specified	117	147985	0.03	0.03	0.04	0.05	0.05	0.06	0.07	0.08	0.09	0.1

G04	Encephalitis, myelitis and encephalomyelitis	158	147944	0.03	0.04	0.04	0.05	0.06	0.07	0.08	0.09	0.11	0.12
G11	Hereditary ataxia	104	147998	0.03	0.03	0.04	0.04	0.05	0.06	0.07	0.08	0.08	0.1
G12	Spinal muscular atrophy and related syndromes	246	147856	0.03	0.04	0.05	0.06	0.07	0.09	0.1	0.12	0.15	0.17
G20	Parkinson's disease	1378	146724	0.04	0.07	0.11	0.16	0.22	0.29	0.38	0.47	0.57	0.66
G23	Other degenerative diseases of basal ganglia	108	147994	0.03	0.03	0.04	0.05	0.05	0.06	0.07	0.08	0.09	0.1
G24	Dystonia	210	147892	0.03	0.04	0.05	0.06	0.07	0.08	0.09	0.11	0.13	0.15
G25	Other extrapyramidal and movement disorders	839	147263	0.04	0.06	0.08	0.11	0.15	0.2	0.25	0.32	0.38	0.46
G30	Alzheimer's disease	1163	146939	0.04	0.06	0.1	0.14	0.19	0.26	0.33	0.41	0.5	0.59
G31	Other degenerative diseases of nervous system, not elsewhere classified	929	147173	0.04	0.06	0.08	0.12	0.16	0.21	0.27	0.34	0.42	0.49
G35	Multiple sclerosis	783	147319	0.04	0.05	0.08	0.11	0.14	0.19	0.24	0.3	0.36	0.43
G37	Other demyelinating diseases of central nervous system	159	147943	0.03	0.04	0.04	0.05	0.06	0.07	0.08	0.09	0.11	0.12
G40	Epilepsy	2411	145691	0.05	0.09	0.15	0.24	0.34	0.46	0.59	0.7	0.8	0.88
G41	Status epilepticus	129	147973	0.03	0.03	0.04	0.05	0.06	0.06	0.07	0.08	0.1	0.11
G43	Migraine	2463	145639	0.05	0.09	0.16	0.24	0.35	0.47	0.6	0.71	0.81	0.88
G44	Other headache syndromes	425	147677	0.03	0.04	0.06	0.08	0.1	0.12	0.15	0.18	0.22	0.26
G45	Transient cerebral ischaemic attacks and related syndromes	1969	146133	0.05	0.08	0.13	0.2	0.29	0.39	0.51	0.62	0.72	0.81
G47	Sleep disorders	3883	144219	0.06	0.12	0.22	0.35	0.5	0.66	0.79	0.88	0.94	0.98
G50	Disorders of trigeminal nerve	376	147726	0.03	0.04	0.06	0.07	0.09	0.11	0.14	0.17	0.2	0.24
G51	Facial nerve disorders	641	147461	0.04	0.05	0.07	0.09	0.12	0.16	0.2	0.25	0.31	0.37
G53	Cranial nerve disorders in diseases classified elsewhere	88	148014	0.03	0.03	0.04	0.04	0.05	0.05	0.06	0.07	0.08	0.09
G54	Nerve root and plexus disorders	252	147850	0.03	0.04	0.05	0.06	0.07	0.09	0.11	0.13	0.15	0.17

G55	Nerve root and plexus compressions in diseases classified elsewhere	3097	145005	0.05	0.1	0.18	0.29	0.42	0.56	0.69	0.81	0.89	0.94
G56	Mononeuropathies of upper limb	5977	142125	0.07	0.16	0.31	0.49	0.68	0.83	0.92	0.97	0.99	1
G57	Mononeuropathies of lower limb	964	147138	0.04	0.06	0.09	0.12	0.17	0.22	0.28	0.35	0.43	0.51
G58	Other mononeuropathies	242	147860	0.03	0.04	0.05	0.06	0.07	0.09	0.1	0.12	0.14	0.17
G59	Mononeuropathy in diseases classified elsewhere	111	147991	0.03	0.03	0.04	0.05	0.05	0.06	0.07	0.08	0.09	0.1
G60	Hereditary and idiopathic neuropathy	128	147974	0.03	0.03	0.04	0.05	0.06	0.06	0.07	0.08	0.1	0.11
G61	Inflammatory polyneuropathy	190	147912	0.03	0.04	0.05	0.05	0.06	0.08	0.09	0.1	0.12	0.14
G62	Other polyneuropathies	1392	146710	0.04	0.07	0.11	0.16	0.22	0.3	0.38	0.48	0.57	0.66
G63	Polyneuropathy in diseases classified elsewhere	440	147662	0.03	0.05	0.06	0.08	0.1	0.12	0.15	0.19	0.23	0.27
G70	Myasthenia gravis and other myoneural disorders	164	147938	0.03	0.04	0.04	0.05	0.06	0.07	0.08	0.1	0.11	0.13
G71	Primary disorders of muscles	101	148001	0.03	0.03	0.04	0.04	0.05	0.06	0.07	0.07	0.08	0.09
G72	Other myopathies	141	147961	0.03	0.04	0.04	0.05	0.06	0.07	0.08	0.09	0.1	0.12
G80	Infantile cerebral palsy	86	148016	0.03	0.03	0.04	0.04	0.05	0.05	0.06	0.07	0.08	0.09
G81	Hemiplegia	1468	146634	0.04	0.07	0.11	0.16	0.23	0.31	0.4	0.5	0.59	0.68
G82	Paraplegia and tetraplegia	234	147868	0.03	0.04	0.05	0.06	0.07	0.08	0.1	0.12	0.14	0.16
G83	Other paralytic syndromes	345	147757	0.03	0.04	0.05	0.07	0.09	0.11	0.13	0.16	0.19	0.22
G90	Disorders of autonomic nervous system	227	147875	0.03	0.04	0.05	0.06	0.07	0.08	0.1	0.12	0.14	0.16
G91	Hydrocephalus	380	147722	0.03	0.04	0.06	0.07	0.09	0.11	0.14	0.17	0.2	0.24
G93	Other disorders of brain	1525	146577	0.04	0.07	0.11	0.17	0.24	0.32	0.41	0.51	0.61	0.7
G95	Other diseases of spinal cord	492	147610	0.03	0.05	0.06	0.08	0.11	0.13	0.17	0.2	0.25	0.29
G96	Other disorders of central nervous system	204	147898	0.03	0.04	0.05	0.06	0.07	0.08	0.09	0.11	0.13	0.15
G97	Postprocedural disorders of nervous system, not elsewhere classified	120	147982	0.03	0.03	0.04	0.05	0.05	0.06	0.07	0.08	0.09	0.1

G99	Other disorders of nervous system in diseases classified elsewhere	898	147204	0.04	0.06	0.08	0.12	0.16	0.21	0.27	0.33	0.41	0.48
H00	Hordeolum and chalazion	969	147133	0.04	0.06	0.09	0.12	0.17	0.22	0.28	0.36	0.43	0.51
H01	Other inflammation of eyelid	583	147519	0.04	0.05	0.07	0.09	0.12	0.15	0.19	0.23	0.28	0.34
H02	Other disorders of eyelid	3684	144418	0.06	0.12	0.21	0.34	0.48	0.63	0.77	0.87	0.93	0.97
H04	Disorders of lachrymal system	1604	146498	0.04	0.07	0.12	0.17	0.25	0.33	0.43	0.53	0.63	0.72
H05	Disorders of orbit	187	147915	0.03	0.04	0.04	0.05	0.06	0.08	0.09	0.1	0.12	0.14
H10	Conjunctivitis	237	147865	0.03	0.04	0.05	0.06	0.07	0.09	0.1	0.12	0.14	0.16
H11	Other disorders of conjunctiva	576	147526	0.04	0.05	0.07	0.09	0.12	0.15	0.19	0.23	0.28	0.33
H16	Keratitis	210	147892	0.03	0.04	0.05	0.06	0.07	0.08	0.09	0.11	0.13	0.15
H17	Corneal scars and opacities	134	147968	0.03	0.04	0.04	0.05	0.06	0.06	0.07	0.09	0.1	0.11
H18	Other disorders of cornea	1226	146876	0.04	0.07	0.1	0.14	0.2	0.27	0.35	0.43	0.52	0.61
H20	Iridocyclitis	292	147810	0.03	0.04	0.05	0.06	0.08	0.1	0.12	0.14	0.16	0.19
H21	Other disorders of iris and ciliary body	331	147771	0.03	0.04	0.05	0.07	0.08	0.1	0.13	0.15	0.18	0.21
H25	Senile cataract	10654	137448	0.09	0.25	0.48	0.72	0.89	0.97	0.99	1	1	1
H26	Other cataract	13462	134640	0.11	0.29	0.57	0.81	0.94	0.99	1	1	1	1
H27	Other disorders of lens	237	147865	0.03	0.04	0.05	0.06	0.07	0.09	0.1	0.12	0.14	0.16
H31	Other disorders of choroid	204	147898	0.03	0.04	0.05	0.06	0.07	0.08	0.09	0.11	0.13	0.15
H33	Retinal detachments and breaks	2071	146031	0.05	0.08	0.14	0.21	0.3	0.41	0.53	0.64	0.74	0.82
H34	Retinal vascular occlusions	527	147575	0.03	0.05	0.06	0.08	0.11	0.14	0.18	0.22	0.26	0.31
H35	Other retinal disorders	4077	144025	0.06	0.12	0.23	0.36	0.52	0.68	0.81	0.9	0.95	0.98
H36	Retinal disorders in diseases classified elsewhere	1236	146866	0.04	0.07	0.1	0.14	0.2	0.27	0.35	0.43	0.52	0.61
H40	Glaucoma	4560	143542	0.06	0.13	0.25	0.4	0.57	0.72	0.85	0.93	0.97	0.99
H43	Disorders of vitreous body	1322	146780	0.04	0.07	0.1	0.15	0.21	0.28	0.37	0.46	0.55	0.64
H44	Disorders of globe	328	147774	0.03	0.04	0.05	0.07	0.08	0.1	0.13	0.15	0.18	0.21

H47	Other disorders of optic [2nd] nerve and visual pathways	318	147784	0.03	0.04	0.05	0.07	0.08	0.1	0.12	0.15	0.18	0.21
H49	Paralytic strabismus	262	147840	0.03	0.04	0.05	0.06	0.07	0.09	0.11	0.13	0.15	0.18
H50	Other strabismus	576	147526	0.04	0.05	0.07	0.09	0.12	0.15	0.19	0.23	0.28	0.33
H52	Disorders of refraction and accommodation	3406	144696	0.06	0.11	0.2	0.31	0.45	0.6	0.73	0.84	0.91	0.96
H53	Visual disturbances	2560	145542	0.05	0.09	0.16	0.25	0.36	0.49	0.61	0.73	0.82	0.89
H54	Blindness and low vision	971	147131	0.04	0.06	0.09	0.12	0.17	0.22	0.29	0.36	0.43	0.51
H55	Nystagmus and other irregular eye movements	189	147913	0.03	0.04	0.04	0.05	0.06	0.08	0.09	0.1	0.12	0.14
H57	Other disorders of eye and adnexa	460	147642	0.03	0.05	0.06	0.08	0.1	0.13	0.16	0.19	0.23	0.28
H59	Postprocedural disorders of eye and adnexa, not elsewhere classified	212	147890	0.03	0.04	0.05	0.06	0.07	0.08	0.1	0.11	0.13	0.15
H60	Otitis externa	364	147738	0.03	0.04	0.06	0.07	0.09	0.11	0.13	0.16	0.19	0.23
H61	Other disorders of external ear	600	147502	0.04	0.05	0.07	0.09	0.12	0.15	0.19	0.24	0.29	0.35
H65	Nonsuppurative otitis media	584	147518	0.04	0.05	0.07	0.09	0.12	0.15	0.19	0.23	0.28	0.34
H66	Suppurative and unspecified otitis media	479	147623	0.03	0.05	0.06	0.08	0.1	0.13	0.16	0.2	0.24	0.29
H69	Other disorders of Eustachian tube	111	147991	0.03	0.03	0.04	0.05	0.05	0.06	0.07	0.08	0.09	0.1
H71	Cholesteatoma of middle ear	218	147884	0.03	0.04	0.05	0.06	0.07	0.08	0.1	0.11	0.13	0.15
H72	Perforation of tympanic membrane	503	147599	0.03	0.05	0.06	0.08	0.11	0.14	0.17	0.21	0.25	0.3
H73	Other disorders of tympanic membrane	189	147913	0.03	0.04	0.04	0.05	0.06	0.08	0.09	0.1	0.12	0.14
H74	Other disorders of middle ear and mastoid	234	147868	0.03	0.04	0.05	0.06	0.07	0.08	0.1	0.12	0.14	0.16
H80	Otosclerosis	169	147933	0.03	0.04	0.04	0.05	0.06	0.07	0.08	0.1	0.11	0.13
H81	Disorders of vestibular function	1084	147018	0.04	0.06	0.09	0.13	0.18	0.24	0.31	0.39	0.47	0.56
H83	Other diseases of inner ear	553	147549	0.04	0.05	0.07	0.09	0.11	0.14	0.18	0.22	0.27	0.32
H90	Conductive and sensorineural hearing loss	1057	147045	0.04	0.06	0.09	0.13	0.18	0.24	0.31	0.38	0.46	0.55
H91	Other hearing loss	3768	144334	0.06	0.12	0.21	0.34	0.49	0.64	0.78	0.87	0.94	0.97
H92	Otalgia and effusion of ear	318	147784	0.03	0.04	0.05	0.07	0.08	0.1	0.12	0.15	0.18	0.21

H93	Other disorders of ear, not elsewhere classified	646	147456	0.04	0.05	0.07	0.1	0.13	0.16	0.21	0.25	0.31	0.37
I05	Rheumatic mitral valve diseases	284	147818	0.03	0.04	0.05	0.06	0.08	0.09	0.11	0.14	0.16	0.19
I07	Rheumatic tricuspid valve diseases	472	147630	0.03	0.05	0.06	0.08	0.1	0.13	0.16	0.2	0.24	0.28
I08	Multiple valve diseases	2621	145481	0.05	0.1	0.16	0.25	0.37	0.5	0.62	0.74	0.83	0.9
I10	Essential (primary) hypertension	51976	96126	0.22	0.65	0.94	1	1	1	1	1	1	1
I11	Hypertensive heart disease	325	147777	0.03	0.04	0.05	0.07	0.08	0.1	0.12	0.15	0.18	0.21
I12	Hypertensive renal disease	709	147393	0.04	0.05	0.07	0.1	0.13	0.17	0.22	0.27	0.33	0.4
I15	Secondary hypertension	83	148019	0.03	0.03	0.04	0.04	0.05	0.05	0.06	0.07	0.08	0.08
I20	Angina pectoris	11145	136957	0.1	0.25	0.5	0.74	0.9	0.97	1	1	1	1
I21	Acute myocardial infarction	6201	141901	0.07	0.17	0.32	0.51	0.69	0.84	0.93	0.98	0.99	1
I22	Subsequent myocardial infarction	340	147762	0.03	0.04	0.05	0.07	0.09	0.11	0.13	0.15	0.18	0.22
I24	Other acute ischaemic heart diseases	1441	146661	0.04	0.07	0.11	0.16	0.23	0.31	0.39	0.49	0.59	0.68
I25	Chronic ischaemic heart disease	17899	130203	0.12	0.36	0.67	0.89	0.98	1	1	1	1	1
I26	Pulmonary embolism	3297	144805	0.06	0.11	0.19	0.31	0.44	0.59	0.72	0.83	0.91	0.95
I27	Other pulmonary heart diseases	968	147134	0.04	0.06	0.09	0.12	0.17	0.22	0.28	0.36	0.43	0.51
I30	Acute pericarditis	135	147967	0.03	0.04	0.04	0.05	0.06	0.07	0.08	0.09	0.1	0.11
I31	Other diseases of pericardium	1138	146964	0.04	0.06	0.09	0.14	0.19	0.25	0.32	0.41	0.49	0.58
I33	Acute and subacute endocarditis	194	147908	0.03	0.04	0.05	0.05	0.06	0.08	0.09	0.11	0.12	0.14
I34	Nonrheumatic mitral valve disorders	2435	145667	0.05	0.09	0.15	0.24	0.35	0.47	0.59	0.71	0.81	0.88
I35	Nonrheumatic aortic valve disorders	2742	145360	0.05	0.1	0.17	0.26	0.38	0.51	0.64	0.76	0.85	0.91
I36	Nonrheumatic tricuspid valve disorders	169	147933	0.03	0.04	0.04	0.05	0.06	0.07	0.08	0.1	0.11	0.13
I37	Pulmonary valve disorders	225	147877	0.03	0.04	0.05	0.06	0.07	0.08	0.1	0.12	0.14	0.16
I38	Endocarditis, valve unspecified	362	147740	0.03	0.04	0.06	0.07	0.09	0.11	0.13	0.16	0.19	0.23
I42	Cardiomyopathy	1155	146947	0.04	0.06	0.1	0.14	0.19	0.25	0.33	0.41	0.5	0.58
I44	Atrioventricular and left bundle-branch block	4212	143890	0.06	0.13	0.23	0.37	0.54	0.69	0.82	0.91	0.96	0.98
I45	Other conduction disorders	2597	145505	0.05	0.09	0.16	0.25	0.37	0.49	0.62	0.73	0.83	0.9

I46	Cardiac arrest	1026	147076	0.04	0.06	0.09	0.13	0.17	0.23	0.3	0.37	0.45	0.53
I47	Paroxysmal tachycardia	2397	145705	0.05	0.09	0.15	0.24	0.34	0.46	0.59	0.7	0.8	0.87
I48	Atrial fibrillation and flutter	13042	135060	0.1	0.29	0.55	0.8	0.94	0.99	1	1	1	1
I49	Other cardiac arrhythmias	2781	145321	0.05	0.1	0.17	0.27	0.39	0.52	0.65	0.76	0.85	0.92
I50	Heart failure	6099	142003	0.07	0.16	0.31	0.5	0.69	0.84	0.93	0.97	0.99	1
I51	Complications and ill-defined descriptions of heart disease	5800	142302	0.07	0.16	0.3	0.48	0.67	0.82	0.92	0.97	0.99	1
I60	Subarachnoid haemorrhage	581	147521	0.04	0.05	0.07	0.09	0.12	0.15	0.19	0.23	0.28	0.34
I61	Intracerebral haemorrhage	751	147351	0.04	0.05	0.08	0.1	0.14	0.18	0.23	0.29	0.35	0.42
I62	Other nontraumatic intracranial haemorrhage	447	147655	0.03	0.05	0.06	0.08	0.1	0.13	0.16	0.19	0.23	0.27
I63	Cerebral infarction	3183	144919	0.05	0.11	0.19	0.3	0.43	0.57	0.71	0.82	0.9	0.95
I64	Stroke, not specified as haemorrhage or infarction	974	147128	0.04	0.06	0.09	0.12	0.17	0.22	0.29	0.36	0.43	0.51
I65	Occlusion and stenosis of precerebral arteries, not resulting in cerebral infarction	964	147138	0.04	0.06	0.09	0.12	0.17	0.22	0.28	0.35	0.43	0.51
I67	Other cerebrovascular diseases	3400	144702	0.06	0.11	0.2	0.31	0.45	0.6	0.73	0.84	0.91	0.96
I69	Sequelae of cerebrovascular disease	1193	146909	0.04	0.06	0.1	0.14	0.2	0.26	0.34	0.42	0.51	0.6
I70	Atherosclerosis	1257	146845	0.04	0.07	0.1	0.15	0.2	0.27	0.35	0.44	0.53	0.62
I71	Aortic aneurysm and dissection	1576	146526	0.04	0.07	0.11	0.17	0.24	0.33	0.42	0.53	0.62	0.72
I72	Other aneurysm	457	147645	0.03	0.05	0.06	0.08	0.1	0.13	0.16	0.19	0.23	0.28
I73	Other peripheral vascular diseases	3154	144948	0.05	0.11	0.19	0.3	0.43	0.57	0.7	0.81	0.89	0.94
I74	Arterial embolism and thrombosis	678	147424	0.04	0.05	0.07	0.1	0.13	0.17	0.21	0.26	0.32	0.38
I77	Other disorders of arteries and arterioles	1097	147005	0.04	0.06	0.09	0.13	0.18	0.24	0.32	0.39	0.48	0.56
I78	Diseases of capillaries	482	147620	0.03	0.05	0.06	0.08	0.1	0.13	0.16	0.2	0.24	0.29
I80	Phlebitis and thrombophlebitis	2906	145196	0.05	0.1	0.17	0.28	0.4	0.54	0.67	0.78	0.87	0.93
I81	Portal vein thrombosis	157	147945	0.03	0.04	0.04	0.05	0.06	0.07	0.08	0.09	0.11	0.12

I82	Other venous embolism and thrombosis	333	147769	0.03	0.04	0.05	0.07	0.08	0.1	0.13	0.15	0.18	0.21
I83	Varicose veins of lower extremities	6190	141912	0.07	0.17	0.32	0.5	0.69	0.84	0.93	0.98	0.99	1
I84	Haemorrhoids	10852	137250	0.09	0.25	0.49	0.73	0.89	0.97	0.99	1	1	1
I85	Oesophageal varices	427	147675	0.03	0.04	0.06	0.08	0.1	0.12	0.15	0.18	0.22	0.26
I86	Varicose veins of other sites	335	147767	0.03	0.04	0.05	0.07	0.08	0.1	0.13	0.15	0.18	0.21
I87	Other disorders of veins	476	147626	0.03	0.05	0.06	0.08	0.1	0.13	0.16	0.2	0.24	0.29
I89	Other non-infective disorders of lymphatic vessels and lymph nodes	790	147312	0.04	0.05	0.08	0.11	0.14	0.19	0.24	0.3	0.37	0.43
I95	Hypotension	6117	141985	0.07	0.16	0.31	0.5	0.69	0.84	0.93	0.98	0.99	1
I97	Postprocedural disorders of circulatory system, not elsewhere classified	136	147966	0.03	0.04	0.04	0.05	0.06	0.07	0.08	0.09	0.1	0.11
I98	Other disorders of circulatory system in diseases classified elsewhere	213	147889	0.03	0.04	0.05	0.06	0.07	0.08	0.1	0.11	0.13	0.15
I99	Other and unspecified disorders of circulatory system	134	147968	0.03	0.04	0.04	0.05	0.06	0.06	0.07	0.09	0.1	0.11
J00	Acute nasopharyngitis [common cold]	300	147802	0.03	0.04	0.05	0.06	0.08	0.1	0.12	0.14	0.17	0.2
J01	Acute sinusitis	106	147996	0.03	0.03	0.04	0.04	0.05	0.06	0.07	0.08	0.09	0.1
J02	Acute pharyngitis	636	147466	0.04	0.05	0.07	0.09	0.12	0.16	0.2	0.25	0.31	0.36
J03	Acute tonsillitis	314	147788	0.03	0.04	0.05	0.07	0.08	0.1	0.12	0.15	0.17	0.2
J04	Acute laryngitis and tracheitis	96	148006	0.03	0.03	0.04	0.04	0.05	0.06	0.06	0.07	0.08	0.09
J06	Acute upper respiratory infections of multiple and unspecified sites	570	147532	0.04	0.05	0.07	0.09	0.12	0.15	0.19	0.23	0.28	0.33
J10	Influenza due to identified influenza virus	497	147605	0.03	0.05	0.06	0.08	0.11	0.13	0.17	0.21	0.25	0.3
J11	Influenza, virus not identified	140	147962	0.03	0.04	0.04	0.05	0.06	0.07	0.08	0.09	0.1	0.11
J12	Viral pneumonia, not elsewhere classified	857	147245	0.04	0.06	0.08	0.11	0.15	0.2	0.26	0.32	0.39	0.46
J13	Pneumonia due to Streptococcus pneumoniae	178	147924	0.03	0.04	0.04	0.05	0.06	0.07	0.09	0.1	0.12	0.13

J15	Bacterial pneumonia, not elsewhere classified	428	147674	0.03	0.04	0.06	0.08	0.1	0.12	0.15	0.18	0.22	0.26
J17	Pneumonia in diseases classified elsewhere	134	147968	0.03	0.04	0.04	0.05	0.06	0.06	0.07	0.09	0.1	0.11
J18	Pneumonia, organism unspecified	9751	138351	0.09	0.23	0.45	0.69	0.86	0.96	0.99	1	1	1
J20	Acute bronchitis	118	147984	0.03	0.03	0.04	0.05	0.05	0.06	0.07	0.08	0.09	0.1
J22	Unspecified acute lower respiratory infection	6190	141912	0.07	0.17	0.32	0.5	0.69	0.84	0.93	0.98	0.99	1
J30	Vasomotor and allergic rhinitis	1562	146540	0.04	0.07	0.11	0.17	0.24	0.33	0.42	0.52	0.62	0.71
J31	Chronic rhinitis, nasopharyngitis and pharyngitis	611	147491	0.04	0.05	0.07	0.09	0.12	0.16	0.2	0.24	0.3	0.35
J32	Chronic sinusitis	1566	146536	0.04	0.07	0.11	0.17	0.24	0.33	0.42	0.52	0.62	0.71
J33	Nasal polyp	1700	146402	0.04	0.08	0.12	0.18	0.26	0.35	0.45	0.56	0.66	0.75
J34	Other disorders of nose and nasal sinuses	3518	144584	0.06	0.11	0.2	0.32	0.47	0.61	0.75	0.85	0.92	0.96
J35	Chronic diseases of tonsils and adenoids	587	147515	0.04	0.05	0.07	0.09	0.12	0.15	0.19	0.24	0.29	0.34
J36	Peritonsillar abscess	238	147864	0.03	0.04	0.05	0.06	0.07	0.09	0.1	0.12	0.14	0.17
J38	Diseases of vocal cords and larynx, not elsewhere classified	1074	147028	0.04	0.06	0.09	0.13	0.18	0.24	0.31	0.39	0.47	0.55
J39	Other diseases of upper respiratory tract	501	147601	0.03	0.05	0.06	0.08	0.11	0.14	0.17	0.21	0.25	0.3
J40	Bronchitis, not specified as acute or chronic	461	147641	0.03	0.05	0.06	0.08	0.1	0.13	0.16	0.19	0.23	0.28
J42	Unspecified chronic bronchitis	206	147896	0.03	0.04	0.05	0.06	0.07	0.08	0.09	0.11	0.13	0.15
J43	Emphysema	1704	146398	0.04	0.08	0.12	0.18	0.26	0.35	0.45	0.56	0.66	0.75
J44	Other chronic obstructive pulmonary disease	7466	140636	0.08	0.19	0.37	0.58	0.77	0.9	0.97	0.99	1	1
J45	Asthma	16080	132022	0.12	0.33	0.63	0.87	0.97	1	1	1	1	1
J46	Status asthmaticus	135	147967	0.03	0.04	0.04	0.05	0.06	0.07	0.08	0.09	0.1	0.11
J47	Bronchiectasis	1937	146165	0.05	0.08	0.13	0.2	0.29	0.39	0.5	0.61	0.71	0.8
J61	Pneumoconiosis due to asbestos and other mineral fibres	195	147907	0.03	0.04	0.05	0.05	0.07	0.08	0.09	0.11	0.12	0.14
J69	Pneumonitis due to solids and liquids	1191	146911	0.04	0.06	0.1	0.14	0.2	0.26	0.34	0.42	0.51	0.6

J80	Adult respiratory distress syndrome	151	147951	0.03	0.04	0.04	0.05	0.06	0.07	0.08	0.09	0.11	0.12
J81	Pulmonary oedema	388	147714	0.03	0.04	0.06	0.07	0.09	0.11	0.14	0.17	0.2	0.24
J84	Other interstitial pulmonary diseases	1297	146805	0.04	0.07	0.1	0.15	0.21	0.28	0.36	0.45	0.54	0.63
J86	Pyothorax	244	147858	0.03	0.04	0.05	0.06	0.07	0.09	0.1	0.12	0.14	0.17
J90	Pleural effusion, not elsewhere classified	5396	142706	0.07	0.15	0.28	0.45	0.64	0.79	0.9	0.96	0.99	1
J91	Pleural effusion in conditions classified elsewhere	188	147914	0.03	0.04	0.04	0.05	0.06	0.08	0.09	0.1	0.12	0.14
J92	Pleural plaque	811	147291	0.04	0.06	0.08	0.11	0.15	0.19	0.25	0.31	0.37	0.44
J93	Pneumothorax	735	147367	0.04	0.05	0.07	0.1	0.14	0.18	0.23	0.28	0.34	0.41
J94	Other pleural conditions	253	147849	0.03	0.04	0.05	0.06	0.07	0.09	0.11	0.13	0.15	0.17
J95	Postprocedural respiratory disorders, not elsewhere classified	296	147806	0.03	0.04	0.05	0.06	0.08	0.1	0.12	0.14	0.17	0.2
J96	Respiratory failure, not elsewhere classified	2818	145284	0.05	0.1	0.17	0.27	0.39	0.52	0.65	0.77	0.86	0.92
J98	Other respiratory disorders	4490	143612	0.06	0.13	0.24	0.39	0.56	0.72	0.84	0.92	0.97	0.99
J99	Respiratory disorders in diseases classified elsewhere	177	147925	0.03	0.04	0.04	0.05	0.06	0.07	0.09	0.1	0.12	0.13
K00	Disorders of tooth development and eruption	297	147805	0.03	0.04	0.05	0.06	0.08	0.1	0.12	0.14	0.17	0.2
K01	Embedded and impacted teeth	877	147225	0.04	0.06	0.08	0.11	0.16	0.2	0.26	0.33	0.4	0.47
K02	Dental caries	1918	146184	0.05	0.08	0.13	0.2	0.29	0.39	0.5	0.61	0.71	0.8
K04	Diseases of pulp and periapical tissues	1343	146759	0.04	0.07	0.1	0.15	0.21	0.29	0.37	0.46	0.56	0.65
K05	Gingivitis and periodontal diseases	495	147607	0.03	0.05	0.06	0.08	0.11	0.13	0.17	0.21	0.25	0.3
K06	Other disorders of gingiva and edentulous alveolar ridge	196	147906	0.03	0.04	0.05	0.05	0.07	0.08	0.09	0.11	0.12	0.14
K07	Dentofacial anomalies [including malocclusion]	343	147759	0.03	0.04	0.05	0.07	0.09	0.11	0.13	0.16	0.19	0.22
K08	Other disorders of teeth and supporting structures	1672	146430	0.04	0.08	0.12	0.18	0.26	0.35	0.45	0.55	0.65	0.74

K09	Cysts of oral region, not elsewhere classified	229	147873	0.03	0.04	0.05	0.06	0.07	0.08	0.1	0.12	0.14	0.16
K10	Other diseases of jaws	241	147861	0.03	0.04	0.05	0.06	0.07	0.09	0.1	0.12	0.14	0.17
K11	Diseases of salivary glands	624	147478	0.04	0.05	0.07	0.09	0.12	0.16	0.2	0.25	0.3	0.36
K12	Stomatitis and related lesions	762	147340	0.04	0.05	0.08	0.1	0.14	0.18	0.23	0.29	0.35	0.42
K13	Other diseases of lip and oral mucosa	1864	146238	0.05	0.08	0.13	0.19	0.28	0.38	0.49	0.59	0.7	0.78
K14	Diseases of tongue	825	147277	0.04	0.06	0.08	0.11	0.15	0.2	0.25	0.31	0.38	0.45
K20	Oesophagitis	5713	142389	0.07	0.16	0.3	0.48	0.66	0.81	0.91	0.97	0.99	1
K21	Gastro-oesophageal reflux disease	18830	129272	0.13	0.38	0.69	0.91	0.98	1	1	1	1	1
K22	Other diseases of oesophagus	7146	140956	0.08	0.18	0.35	0.56	0.75	0.89	0.96	0.99	1	1
K25	Gastric ulcer	2963	145139	0.05	0.1	0.18	0.28	0.41	0.54	0.68	0.79	0.87	0.93
K26	Duodenal ulcer	1981	146121	0.05	0.08	0.13	0.2	0.29	0.4	0.51	0.62	0.72	0.81
K27	Peptic ulcer, site unspecified	283	147819	0.03	0.04	0.05	0.06	0.08	0.09	0.11	0.14	0.16	0.19
K29	Gastritis and duodenitis	19152	128950	0.13	0.38	0.7	0.91	0.99	1	1	1	1	1
K30	Dyspepsia	6153	141949	0.07	0.16	0.31	0.5	0.69	0.84	0.93	0.98	0.99	1
K31	Other diseases of stomach and duodenum	6373	141729	0.07	0.17	0.32	0.52	0.71	0.85	0.94	0.98	0.99	1
K35	Acute appendicitis	1813	146289	0.05	0.08	0.13	0.19	0.27	0.37	0.47	0.58	0.68	0.77
K37	Unspecified appendicitis	256	147846	0.03	0.04	0.05	0.06	0.07	0.09	0.11	0.13	0.15	0.17
K38	Other diseases of appendix	209	147893	0.03	0.04	0.05	0.06	0.07	0.08	0.09	0.11	0.13	0.15
K40	Inguinal hernia	9614	138488	0.09	0.23	0.44	0.68	0.86	0.95	0.99	1	1	1
K41	Femoral hernia	450	147652	0.03	0.05	0.06	0.08	0.1	0.13	0.16	0.19	0.23	0.27
K42	Umbilical hernia	2478	145624	0.05	0.09	0.16	0.24	0.35	0.47	0.6	0.72	0.81	0.89
K43	Ventral hernia	2145	145957	0.05	0.09	0.14	0.22	0.31	0.42	0.54	0.65	0.75	0.84
K44	Diaphragmatic hernia	18516	129586	0.13	0.37	0.69	0.9	0.98	1	1	1	1	1
K45	Other abdominal hernia	94	148008	0.03	0.03	0.04	0.04	0.05	0.06	0.06	0.07	0.08	0.09
K46	Unspecified abdominal hernia	305	147797	0.03	0.04	0.05	0.07	0.08	0.1	0.12	0.14	0.17	0.2
K50	Crohn's disease [regional enteritis]	1016	147086	0.04	0.06	0.09	0.13	0.17	0.23	0.3	0.37	0.45	0.53

K51	Ulcerative colitis	1810	146292	0.05	0.08	0.13	0.19	0.27	0.37	0.47	0.58	0.68	0.77
K52	Other non-infective gastro-enteritis and colitis	8772	139330	0.08	0.21	0.41	0.64	0.83	0.94	0.98	1	1	1
K55	Vascular disorders of intestine	1091	147011	0.04	0.06	0.09	0.13	0.18	0.24	0.31	0.39	0.47	0.56
K56	Paralytic ileus and intestinal obstruction without hernia	3397	144705	0.06	0.11	0.2	0.31	0.45	0.6	0.73	0.84	0.91	0.96
K57	Diverticular disease of intestine	22706	125396	0.14	0.43	0.76	0.94	0.99	1	1	1	1	1
K58	Irritable bowel syndrome	4201	143901	0.06	0.13	0.23	0.37	0.53	0.69	0.82	0.91	0.96	0.98
K59	Other functional intestinal disorders	10519	137583	0.09	0.24	0.48	0.72	0.89	0.97	0.99	1	1	1
K60	Fissure and fistula of anal and rectal regions	1710	146392	0.04	0.08	0.12	0.18	0.26	0.35	0.45	0.56	0.66	0.75
K61	Abscess of anal and rectal regions	714	147388	0.04	0.05	0.07	0.1	0.13	0.17	0.22	0.28	0.34	0.4
K62	Other diseases of anus and rectum	13741	134361	0.11	0.3	0.57	0.82	0.95	0.99	1	1	1	1
K63	Other diseases of intestine	11901	136201	0.1	0.27	0.52	0.76	0.92	0.98	1	1	1	1
K64	Haemorrhoids and perianal venous thrombosis	6561	141541	0.07	0.17	0.33	0.53	0.72	0.86	0.94	0.98	1	1
K65	Peritonitis	692	147410	0.04	0.05	0.07	0.1	0.13	0.17	0.22	0.27	0.33	0.39
K66	Other disorders of peritoneum	3014	145088	0.05	0.1	0.18	0.28	0.41	0.55	0.68	0.79	0.88	0.94
K70	Alcoholic liver disease	635	147467	0.04	0.05	0.07	0.09	0.12	0.16	0.2	0.25	0.3	0.36
K72	Hepatic failure, not elsewhere classified	422	147680	0.03	0.04	0.06	0.08	0.1	0.12	0.15	0.18	0.22	0.26
K73	Chronic hepatitis, not elsewhere classified	86	148016	0.03	0.03	0.04	0.04	0.05	0.05	0.06	0.07	0.08	0.09
K74	Fibrosis and cirrhosis of liver	846	147256	0.04	0.06	0.08	0.11	0.15	0.2	0.25	0.32	0.39	0.46
K75	Other inflammatory liver diseases	650	147452	0.04	0.05	0.07	0.1	0.13	0.16	0.21	0.26	0.31	0.37
K76	Other diseases of liver	3913	144189	0.06	0.12	0.22	0.35	0.51	0.66	0.79	0.89	0.94	0.98
K80	Cholelithiasis	9355	138747	0.09	0.22	0.44	0.67	0.85	0.95	0.99	1	1	1
K81	Cholecystitis	1780	146322	0.05	0.08	0.12	0.19	0.27	0.36	0.47	0.57	0.68	0.77
K82	Other diseases of gallbladder	1298	146804	0.04	0.07	0.1	0.15	0.21	0.28	0.36	0.45	0.54	0.63
K83	Other diseases of biliary tract	1816	146286	0.05	0.08	0.13	0.19	0.27	0.37	0.48	0.58	0.68	0.77
K85	Acute pancreatitis	1400	146702	0.04	0.07	0.11	0.16	0.22	0.3	0.39	0.48	0.57	0.66

K86	Other diseases of pancreas	1110	146992	0.04	0.06	0.09	0.13	0.18	0.25	0.32	0.4	0.48	0.57
K90	Intestinal malabsorption	1324	146778	0.04	0.07	0.1	0.15	0.21	0.28	0.37	0.46	0.55	0.64
K91	Postprocedural disorders of digestive system, not elsewhere classified	1212	146890	0.04	0.06	0.1	0.14	0.2	0.27	0.34	0.43	0.52	0.6
K92	Other diseases of digestive system	7664	140438	0.08	0.19	0.37	0.59	0.78	0.91	0.97	0.99	1	1
L02	Cutaneous abscess, furuncle and carbuncle	1387	146715	0.04	0.07	0.11	0.16	0.22	0.3	0.38	0.48	0.57	0.66
L03	Cellulitis	5311	142791	0.07	0.15	0.28	0.45	0.63	0.78	0.89	0.96	0.98	1
L05	Pilonidal cyst	280	147822	0.03	0.04	0.05	0.06	0.08	0.09	0.11	0.13	0.16	0.19
L08	Other local infections of skin and subcutaneous tissue	1124	146978	0.04	0.06	0.09	0.13	0.19	0.25	0.32	0.4	0.49	0.57
L20	Atopic dermatitis	97	148005	0.03	0.03	0.04	0.04	0.05	0.06	0.06	0.07	0.08	0.09
L24	Irritant contact dermatitis	258	147844	0.03	0.04	0.05	0.06	0.07	0.09	0.11	0.13	0.15	0.18
L27	Dermatitis due to substances taken internally	395	147707	0.03	0.04	0.06	0.07	0.09	0.12	0.14	0.17	0.21	0.25
L28	Lichen simplex chronicus and prurigo	164	147938	0.03	0.04	0.04	0.05	0.06	0.07	0.08	0.1	0.11	0.13
L29	Pruritus	565	147537	0.04	0.05	0.07	0.09	0.11	0.15	0.18	0.23	0.28	0.33
L30	Other dermatitis	1631	146471	0.04	0.07	0.12	0.18	0.25	0.34	0.44	0.54	0.64	0.73
L40	Psoriasis	1930	146172	0.05	0.08	0.13	0.2	0.29	0.39	0.5	0.61	0.71	0.8
L43	Lichen planus	421	147681	0.03	0.04	0.06	0.08	0.1	0.12	0.15	0.18	0.22	0.26
L50	Urticaria	270	147832	0.03	0.04	0.05	0.06	0.08	0.09	0.11	0.13	0.16	0.18
L53	Other erythematous conditions	475	147627	0.03	0.05	0.06	0.08	0.1	0.13	0.16	0.2	0.24	0.29
L57	Skin changes due to chronic exposure to nonionising radiation	2156	145946	0.05	0.09	0.14	0.22	0.31	0.42	0.54	0.66	0.76	0.84
L60	Nail disorders	722	147380	0.04	0.05	0.07	0.1	0.14	0.18	0.22	0.28	0.34	0.4
L65	Other nonscarring hair loss	79	148023	0.03	0.03	0.04	0.04	0.05	0.05	0.06	0.07	0.07	0.08
L71	Rosacea	287	147815	0.03	0.04	0.05	0.06	0.08	0.1	0.11	0.14	0.16	0.19
L72	Follicular cysts of skin and subcutaneous tissue	4511	143591	0.06	0.13	0.25	0.4	0.56	0.72	0.84	0.92	0.97	0.99

L73	Other follicular disorders	248	147854	0.03	0.04	0.05	0.06	0.07	0.09	0.1	0.12	0.15	0.17
L81	Other disorders of pigmentation	568	147534	0.04	0.05	0.07	0.09	0.12	0.15	0.19	0.23	0.28	0.33
L82	Seborrhoeic keratosis	2213	145889	0.05	0.09	0.14	0.22	0.32	0.43	0.55	0.67	0.77	0.85
L84	Corns and callosities	192	147910	0.03	0.04	0.05	0.05	0.06	0.08	0.09	0.11	0.12	0.14
L85	Other epidermal thickening	567	147535	0.04	0.05	0.07	0.09	0.12	0.15	0.19	0.23	0.28	0.33
L89	Decubitus ulcer	1680	146422	0.04	0.08	0.12	0.18	0.26	0.35	0.45	0.55	0.65	0.74
L90	Atrophic disorders of skin	1668	146434	0.04	0.07	0.12	0.18	0.25	0.34	0.44	0.55	0.65	0.74
L91	Hypertrophic disorders of skin	569	147533	0.04	0.05	0.07	0.09	0.12	0.15	0.19	0.23	0.28	0.33
L92	Granulomatous disorders of skin and subcutaneous tissue	327	147775	0.03	0.04	0.05	0.07	0.08	0.1	0.12	0.15	0.18	0.21
L93	Lupus erythematosus	90	148012	0.03	0.03	0.04	0.04	0.05	0.06	0.06	0.07	0.08	0.09
L94	Other localised connective tissue disorders	93	148009	0.03	0.03	0.04	0.04	0.05	0.06	0.06	0.07	0.08	0.09
L95	Vasculitis limited to skin, not elsewhere classified	93	148009	0.03	0.03	0.04	0.04	0.05	0.06	0.06	0.07	0.08	0.09
L97	Ulcer of lower limb, not elsewhere classified	1184	146918	0.04	0.06	0.1	0.14	0.19	0.26	0.34	0.42	0.51	0.59
L98	Other disorders of skin and subcutaneous tissue, not elsewhere classified	4240	143862	0.06	0.13	0.23	0.38	0.54	0.69	0.82	0.91	0.96	0.98
M00	Pyogenic arthritis	298	147804	0.03	0.04	0.05	0.06	0.08	0.1	0.12	0.14	0.17	0.2
M05	Seropositive rheumatoid arthritis	446	147656	0.03	0.05	0.06	0.08	0.1	0.13	0.16	0.19	0.23	0.27
M06	Other rheumatoid arthritis	3165	144937	0.05	0.11	0.19	0.3	0.43	0.57	0.7	0.81	0.89	0.95
M07	Psoriatic and enteropathic arthropathies	510	147592	0.03	0.05	0.06	0.08	0.11	0.14	0.17	0.21	0.25	0.3
M10	Gout	3384	144718	0.06	0.11	0.2	0.31	0.45	0.6	0.73	0.84	0.91	0.96
M11	Other crystal arthropathies	345	147757	0.03	0.04	0.05	0.07	0.09	0.11	0.13	0.16	0.19	0.22
M12	Other specific arthropathies	112	147990	0.03	0.03	0.04	0.05	0.05	0.06	0.07	0.08	0.09	0.1
M13	Other arthritis	10795	137307	0.09	0.25	0.48	0.73	0.89	0.97	0.99	1	1	1
M15	Polyarthrosis	4082	144020	0.06	0.12	0.23	0.36	0.52	0.68	0.81	0.9	0.95	0.98

M16	Coxarthrosis [arthrosis of hip]	8934	139168	0.08	0.22	0.42	0.65	0.83	0.94	0.98	1	1	1
M17	Gonarthrosis [arthrosis of knee]	13973	134129	0.11	0.3	0.58	0.82	0.95	0.99	1	1	1	1
M18	Arthrosis of first carpometacarpal joint	1000	147102	0.04	0.06	0.09	0.12	0.17	0.23	0.29	0.36	0.44	0.52
M19	Other arthrosis	15690	132412	0.11	0.33	0.62	0.86	0.97	1	1	1	1	1
M20	Acquired deformities of fingers and toes	5365	142737	0.07	0.15	0.28	0.45	0.63	0.79	0.9	0.96	0.99	1
M21	Other acquired deformities of limbs	1520	146582	0.04	0.07	0.11	0.17	0.24	0.32	0.41	0.51	0.61	0.7
M22	Disorders of patella	489	147613	0.03	0.05	0.06	0.08	0.11	0.13	0.17	0.2	0.25	0.29
M23	Internal derangement of knee	7786	140316	0.08	0.19	0.38	0.59	0.79	0.91	0.97	0.99	1	1
M24	Other specific joint derangements	1577	146525	0.04	0.07	0.11	0.17	0.24	0.33	0.42	0.53	0.62	0.72
M25	Other joint disorders, not elsewhere classified	9216	138886	0.09	0.22	0.43	0.66	0.85	0.95	0.99	1	1	1
M31	Other necrotising vasculopathies	539	147563	0.04	0.05	0.06	0.09	0.11	0.14	0.18	0.22	0.27	0.32
M32	Systemic lupus erythematosus	219	147883	0.03	0.04	0.05	0.06	0.07	0.08	0.1	0.11	0.13	0.16
M34	Systemic sclerosis	122	147980	0.03	0.03	0.04	0.05	0.05	0.06	0.07	0.08	0.09	0.11
M35	Other systemic involvement of connective tissue	2035	146067	0.05	0.08	0.14	0.21	0.3	0.41	0.52	0.63	0.73	0.82
M40	Kyphosis and lordosis	288	147814	0.03	0.04	0.05	0.06	0.08	0.1	0.12	0.14	0.16	0.19
M41	Scoliosis	1146	146956	0.04	0.06	0.09	0.14	0.19	0.25	0.33	0.41	0.49	0.58
M43	Other deforming dorsopathies	1533	146569	0.04	0.07	0.11	0.17	0.24	0.32	0.42	0.51	0.61	0.7
M45	Ankylosing spondylitis	416	147686	0.03	0.04	0.06	0.08	0.1	0.12	0.15	0.18	0.22	0.26
M46	Other inflammatory spondylopathies	1244	146858	0.04	0.07	0.1	0.14	0.2	0.27	0.35	0.44	0.53	0.61
M47	Spondylosis	6853	141249	0.07	0.18	0.34	0.54	0.74	0.87	0.95	0.99	1	1
M48	Other spondylopathies	3923	144179	0.06	0.12	0.22	0.35	0.51	0.66	0.79	0.89	0.95	0.98
M49	Spondylopathies in diseases classified elsewhere	138	147964	0.03	0.04	0.04	0.05	0.06	0.07	0.08	0.09	0.1	0.11
M50	Cervical disk disorders	1150	146952	0.04	0.06	0.09	0.14	0.19	0.25	0.33	0.41	0.49	0.58
M51	Other intervertebral disk disorders	5419	142683	0.07	0.15	0.28	0.46	0.64	0.79	0.9	0.96	0.99	1

M53	Other dorsopathies, not elsewhere classified	385	147717	0.03	0.04	0.06	0.07	0.09	0.11	0.14	0.17	0.2	0.24
M54	Dorsalgia	10822	137280	0.09	0.25	0.49	0.73	0.89	0.97	0.99	1	1	1
M60	Myositis	170	147932	0.03	0.04	0.04	0.05	0.06	0.07	0.08	0.1	0.11	0.13
M62	Other disorders of muscle	774	147328	0.04	0.05	0.08	0.11	0.14	0.19	0.24	0.3	0.36	0.43
M65	Synovitis and tenosynovitis	3193	144909	0.05	0.11	0.19	0.3	0.43	0.57	0.71	0.82	0.9	0.95
M66	Spontaneous rupture of synovium and tendon	347	147755	0.03	0.04	0.05	0.07	0.09	0.11	0.13	0.16	0.19	0.22
M67	Other disorders of synovium and tendon	2219	145883	0.05	0.09	0.14	0.22	0.32	0.43	0.55	0.67	0.77	0.85
M70	Soft tissue disorders related to use, overuse and pressure	1068	147034	0.04	0.06	0.09	0.13	0.18	0.24	0.31	0.39	0.47	0.55
M71	Other bursopathies	629	147473	0.04	0.05	0.07	0.09	0.12	0.16	0.2	0.25	0.3	0.36
M72	Fibroblastic disorders	2590	145512	0.05	0.09	0.16	0.25	0.37	0.49	0.62	0.73	0.83	0.9
M75	Shoulder lesions	5357	142745	0.07	0.15	0.28	0.45	0.63	0.79	0.9	0.96	0.99	1
M76	Enthesopathies of lower limb, excluding foot	332	147770	0.03	0.04	0.05	0.07	0.08	0.1	0.13	0.15	0.18	0.21
M77	Other enthesopathies	1039	147063	0.04	0.06	0.09	0.13	0.18	0.23	0.3	0.38	0.46	0.54
M79	Other soft tissue disorders, not elsewhere classified	9102	139000	0.09	0.22	0.43	0.66	0.84	0.94	0.99	1	1	1
M80	Osteoporosis with pathological fracture	726	147376	0.04	0.05	0.07	0.1	0.14	0.18	0.23	0.28	0.34	0.41
M81	Osteoporosis without pathological fracture	6033	142069	0.07	0.16	0.31	0.5	0.68	0.83	0.93	0.97	0.99	1
M84	Disorders of continuity of bone	1035	147067	0.04	0.06	0.09	0.13	0.18	0.23	0.3	0.38	0.46	0.54
M85	Other disorders of bone density and structure	1928	146174	0.05	0.08	0.13	0.2	0.29	0.39	0.5	0.61	0.71	0.8
M86	Osteomyelitis	468	147634	0.03	0.05	0.06	0.08	0.1	0.13	0.16	0.2	0.24	0.28
M87	Osteonecrosis	386	147716	0.03	0.04	0.06	0.07	0.09	0.11	0.14	0.17	0.2	0.24
M88	Paget's disease of bone [osteitis deformans]	138	147964	0.03	0.04	0.04	0.05	0.06	0.07	0.08	0.09	0.1	0.11
M89	Other disorders of bone	969	147133	0.04	0.06	0.09	0.12	0.17	0.22	0.28	0.36	0.43	0.51
M90	Osteopathies in diseases classified elsewhere	331	147771	0.03	0.04	0.05	0.07	0.08	0.1	0.13	0.15	0.18	0.21
M93	Other osteochondropathies	159	147943	0.03	0.04	0.04	0.05	0.06	0.07	0.08	0.09	0.11	0.12

M94	Other disorders of cartilage	512	147590	0.03	0.05	0.06	0.08	0.11	0.14	0.17	0.21	0.26	0.3
M95	Other acquired deformities of musculoskeletal system and connective tissue	155	147947	0.03	0.04	0.04	0.05	0.06	0.07	0.08	0.09	0.11	0.12
M96	Postprocedural musculoskeletal disorders, not elsewhere classified	433	147669	0.03	0.05	0.06	0.08	0.1	0.12	0.15	0.19	0.22	0.26
M99	Biomechanical lesions, not elsewhere classified	170	147932	0.03	0.04	0.04	0.05	0.06	0.07	0.08	0.1	0.11	0.13
N02	Recurrent and persistent haematuria	381	147721	0.03	0.04	0.06	0.07	0.09	0.11	0.14	0.17	0.2	0.24
N03	Chronic nephritic syndrome	394	147708	0.03	0.04	0.06	0.07	0.09	0.12	0.14	0.17	0.21	0.24
N04	Nephrotic syndrome	139	147963	0.03	0.04	0.04	0.05	0.06	0.07	0.08	0.09	0.1	0.11
N05	Unspecified nephritic syndrome	163	147939	0.03	0.04	0.04	0.05	0.06	0.07	0.08	0.1	0.11	0.13
N08	Glomerular disorders in diseases classified elsewhere	305	147797	0.03	0.04	0.05	0.07	0.08	0.1	0.12	0.14	0.17	0.2
N10	Acute tubulo-interstitial nephritis	139	147963	0.03	0.04	0.04	0.05	0.06	0.07	0.08	0.09	0.1	0.11
N11	Chronic tubulo-interstitial nephritis	106	147996	0.03	0.03	0.04	0.04	0.05	0.06	0.07	0.08	0.09	0.1
N12	Tubulo-interstitial nephritis, not specified as acute or chronic	745	147357	0.04	0.05	0.08	0.1	0.14	0.18	0.23	0.29	0.35	0.41
N13	Obstructive and reflux uropathy	2421	145681	0.05	0.09	0.15	0.24	0.35	0.47	0.59	0.71	0.8	0.88
N17	Acute renal failure	7667	140435	0.08	0.19	0.37	0.59	0.78	0.91	0.97	0.99	1	1
N18	Chronic renal failure	7066	141036	0.08	0.18	0.35	0.56	0.75	0.88	0.96	0.99	1	1
N19	Unspecified renal failure	941	147161	0.04	0.06	0.08	0.12	0.16	0.22	0.28	0.35	0.42	0.5
N20	Calculus of kidney and ureter	3162	144940	0.05	0.11	0.19	0.3	0.43	0.57	0.7	0.81	0.89	0.95
N21	Calculus of lower urinary tract	637	147465	0.04	0.05	0.07	0.09	0.12	0.16	0.2	0.25	0.31	0.36
N23	Unspecified renal colic	925	147177	0.04	0.06	0.08	0.12	0.16	0.21	0.27	0.34	0.42	0.49
N26	Unspecified contracted kidney	146	147956	0.03	0.04	0.04	0.05	0.06	0.07	0.08	0.09	0.1	0.12
N28	Other disorders of kidney and ureter, not elsewhere classified	2543	145559	0.05	0.09	0.16	0.25	0.36	0.48	0.61	0.73	0.82	0.89

N30	Cystitis	1884	146218	0.05	0.08	0.13	0.2	0.28	0.38	0.49	0.6	0.7	0.79
N31	Neuromuscular dysfunction of bladder, not elsewhere classified	834	147268	0.04	0.06	0.08	0.11	0.15	0.2	0.25	0.31	0.38	0.45
N32	Other disorders of bladder	5807	142295	0.07	0.16	0.3	0.48	0.67	0.82	0.92	0.97	0.99	1
N34	Urethritis and urethral syndrome	93	148009	0.03	0.03	0.04	0.04	0.05	0.06	0.06	0.07	0.08	0.09
N35	Urethral stricture	2034	146068	0.05	0.08	0.14	0.21	0.3	0.41	0.52	0.63	0.73	0.82
N36	Other disorders of urethra	503	147599	0.03	0.05	0.06	0.08	0.11	0.14	0.17	0.21	0.25	0.3
N39	Other disorders of urinary system	13197	134905	0.1	0.29	0.56	0.8	0.94	0.99	1	1	1	1
N40	Hyperplasia of prostate	9293	138809	0.09	0.22	0.43	0.67	0.85	0.95	0.99	1	1	1
N41	Inflammatory diseases of prostate	1060	147042	0.04	0.06	0.09	0.13	0.18	0.24	0.31	0.38	0.46	0.55
N42	Other disorders of prostate	879	147223	0.04	0.06	0.08	0.11	0.16	0.2	0.26	0.33	0.4	0.47
N43	Hydrocele and spermatocele	903	147199	0.04	0.06	0.08	0.12	0.16	0.21	0.27	0.34	0.41	0.48
N45	Orchitis and epididymitis	553	147549	0.04	0.05	0.07	0.09	0.11	0.14	0.18	0.22	0.27	0.32
N47	Redundant prepuce, phimosis and paraphimosis	1335	146767	0.04	0.07	0.1	0.15	0.21	0.29	0.37	0.46	0.55	0.64
N48	Other disorders of penis	1357	146745	0.04	0.07	0.1	0.15	0.22	0.29	0.38	0.47	0.56	0.65
N49	Inflammatory disorders of male genital organs, not elsewhere classified	108	147994	0.03	0.03	0.04	0.05	0.05	0.06	0.07	0.08	0.09	0.1
N50	Other disorders of male genital organs	1656	146446	0.04	0.07	0.12	0.18	0.25	0.34	0.44	0.54	0.65	0.74
N60	Benign mammary dysplasia	1024	147078	0.04	0.06	0.09	0.13	0.17	0.23	0.3	0.37	0.45	0.53
N61	Inflammatory disorders of breast	277	147825	0.03	0.04	0.05	0.06	0.08	0.09	0.11	0.13	0.16	0.19
N62	Hypertrophy of breast	404	147698	0.03	0.04	0.06	0.07	0.09	0.12	0.14	0.18	0.21	0.25
N63	Unspecified lump in breast	807	147295	0.04	0.06	0.08	0.11	0.15	0.19	0.25	0.31	0.37	0.44
N64	Other disorders of breast	713	147389	0.04	0.05	0.07	0.1	0.13	0.17	0.22	0.28	0.34	0.4
N70	Salpingitis and oophoritis	323	147779	0.03	0.04	0.05	0.07	0.08	0.1	0.12	0.15	0.18	0.21
N71	Inflammatory disease of uterus, except cervix	131	147971	0.03	0.03	0.04	0.05	0.06	0.06	0.07	0.08	0.1	0.11
N72	Inflammatory disease of cervix uteri	598	147504	0.04	0.05	0.07	0.09	0.12	0.15	0.19	0.24	0.29	0.35

N73	Other female pelvic inflammatory diseases	1441	146661	0.04	0.07	0.11	0.16	0.23	0.31	0.39	0.49	0.59	0.68
N75	Diseases of Bartholin's gland	351	147751	0.03	0.04	0.05	0.07	0.09	0.11	0.13	0.16	0.19	0.22
N76	Other inflammation of vagina and vulva	454	147648	0.03	0.05	0.06	0.08	0.1	0.13	0.16	0.19	0.23	0.28
N77	Vulvovaginal ulceration and inflammation in diseases classified elsewhere	148	147954	0.03	0.04	0.04	0.05	0.06	0.07	0.08	0.09	0.1	0.12
N80	Endometriosis	1938	146164	0.05	0.08	0.13	0.2	0.29	0.39	0.5	0.61	0.71	0.8
N81	Female genital prolapse	6162	141940	0.07	0.16	0.31	0.5	0.69	0.84	0.93	0.98	0.99	1
N82	Fistulae involving female genital tract	107	147995	0.03	0.03	0.04	0.05	0.05	0.06	0.07	0.08	0.09	0.1
N83	Noninflammatory disorders of ovary, Fallopian tube and broad ligament	2772	145330	0.05	0.1	0.17	0.27	0.39	0.52	0.65	0.76	0.85	0.92
N84	Polyp of female genital tract	5286	142816	0.07	0.15	0.28	0.45	0.63	0.78	0.89	0.96	0.98	1
N85	Other noninflammatory disorders of uterus, except cervix	2677	145425	0.05	0.1	0.16	0.26	0.38	0.5	0.63	0.75	0.84	0.91
N86	Erosion and ectropion of cervix uteri	486	147616	0.03	0.05	0.06	0.08	0.1	0.13	0.17	0.2	0.24	0.29
N87	Dysplasia of cervix uteri	700	147402	0.04	0.05	0.07	0.1	0.13	0.17	0.22	0.27	0.33	0.39
N88	Other noninflammatory disorders of cervix uteri	976	147126	0.04	0.06	0.09	0.12	0.17	0.22	0.29	0.36	0.43	0.51
N89	Other noninflammatory disorders of vagina	883	147219	0.04	0.06	0.08	0.11	0.16	0.21	0.26	0.33	0.4	0.47
N90	Other noninflammatory disorders of vulva and perineum	1115	146987	0.04	0.06	0.09	0.13	0.19	0.25	0.32	0.4	0.48	0.57
N92	Excessive, frequent and irregular menstruation	6373	141729	0.07	0.17	0.32	0.52	0.71	0.85	0.94	0.98	0.99	1
N93	Other abnormal uterine and vaginal bleeding	1956	146146	0.05	0.08	0.13	0.2	0.29	0.39	0.5	0.61	0.72	0.8
N94	Pain and other conditions associated with female genital organs and menstrual cycle	1499	146603	0.04	0.07	0.11	0.17	0.23	0.32	0.41	0.51	0.6	0.69
N95	Menopausal and other perimenopausal disorders	5292	142810	0.07	0.15	0.28	0.45	0.63	0.78	0.89	0.96	0.98	1
N97	Female infertility	576	147526	0.04	0.05	0.07	0.09	0.12	0.15	0.19	0.23	0.28	0.33

N99	Postprocedural disorders of genito-urinary system, not elsewhere classified	667	147435	0.04	0.05	0.07	0.1	0.13	0.17	0.21	0.26	0.32	0.38
O00	Ectopic pregnancy	139	147963	0.03	0.04	0.04	0.05	0.06	0.07	0.08	0.09	0.1	0.11
O02	Other abnormal products of conception	603	147499	0.04	0.05	0.07	0.09	0.12	0.15	0.19	0.24	0.29	0.35
O03	Spontaneous abortion	605	147497	0.04	0.05	0.07	0.09	0.12	0.15	0.19	0.24	0.29	0.35
O04	Medical abortion	672	147430	0.04	0.05	0.07	0.1	0.13	0.17	0.21	0.26	0.32	0.38
O06	Unspecified abortion	133	147969	0.03	0.03	0.04	0.05	0.06	0.06	0.07	0.09	0.1	0.11
O13	Gestational [pregnancy-induced] hypertension without significant proteinuria	180	147922	0.03	0.04	0.04	0.05	0.06	0.07	0.09	0.1	0.12	0.14
O14	Gestational [pregnancy-induced] hypertension with significant proteinuria	119	147983	0.03	0.03	0.04	0.05	0.05	0.06	0.07	0.08	0.09	0.1
O16	Unspecified maternal hypertension	227	147875	0.03	0.04	0.05	0.06	0.07	0.08	0.1	0.12	0.14	0.16
O20	Haemorrhage in early pregnancy	442	147660	0.03	0.05	0.06	0.08	0.1	0.12	0.15	0.19	0.23	0.27
O21	Excessive vomiting in pregnancy	81	148021	0.03	0.03	0.04	0.04	0.05	0.05	0.06	0.07	0.07	0.08
O23	Infections of genito-urinary tract in pregnancy	93	148009	0.03	0.03	0.04	0.04	0.05	0.06	0.06	0.07	0.08	0.09
O24	Diabetes mellitus in pregnancy	77	148025	0.03	0.03	0.04	0.04	0.05	0.05	0.06	0.07	0.07	0.08
O26	Maternal care for other conditions predominantly related to pregnancy	632	147470	0.04	0.05	0.07	0.09	0.12	0.16	0.2	0.25	0.3	0.36
O30	Multiple gestation	95	148007	0.03	0.03	0.04	0.04	0.05	0.06	0.06	0.07	0.08	0.09
O32	Maternal care for known or suspected malpresentation of foetus	412	147690	0.03	0.04	0.06	0.07	0.09	0.12	0.15	0.18	0.21	0.25
O34	Maternal care for known or suspected abnormality of pelvic organs	575	147527	0.04	0.05	0.07	0.09	0.12	0.15	0.19	0.23	0.28	0.33
O35	Maternal care for known or suspected foetal abnormality and damage	115	147987	0.03	0.03	0.04	0.05	0.05	0.06	0.07	0.08	0.09	0.1

O36	Maternal care for other known or suspected foetal problems	721	147381	0.04	0.05	0.07	0.1	0.14	0.18	0.22	0.28	0.34	0.4
O41	Other disorders of amniotic fluid and membranes	96	148006	0.03	0.03	0.04	0.04	0.05	0.06	0.06	0.07	0.08	0.09
O42	Premature rupture of membranes	403	147699	0.03	0.04	0.06	0.07	0.09	0.12	0.14	0.18	0.21	0.25
O43	Placental disorders	84	148018	0.03	0.03	0.04	0.04	0.05	0.05	0.06	0.07	0.08	0.08
O46	Antepartum haemorrhage, not elsewhere classified	240	147862	0.03	0.04	0.05	0.06	0.07	0.09	0.1	0.12	0.14	0.17
O47	False labour	363	147739	0.03	0.04	0.06	0.07	0.09	0.11	0.13	0.16	0.19	0.23
O48	Prolonged pregnancy	473	147629	0.03	0.05	0.06	0.08	0.1	0.13	0.16	0.2	0.24	0.28
O60	Preterm delivery	246	147856	0.03	0.04	0.05	0.06	0.07	0.09	0.1	0.12	0.15	0.17
O62	Abnormalities of forces of labour	182	147920	0.03	0.04	0.04	0.05	0.06	0.07	0.09	0.1	0.12	0.14
O63	Long labour	677	147425	0.04	0.05	0.07	0.1	0.13	0.17	0.21	0.26	0.32	0.38
O64	Obstructed labour due to malposition and malpresentation of foetus	140	147962	0.03	0.04	0.04	0.05	0.06	0.07	0.08	0.09	0.1	0.11
O66	Other obstructed labour	113	147989	0.03	0.03	0.04	0.05	0.05	0.06	0.07	0.08	0.09	0.1
O68	Labour and delivery complicated by foetal stress [distress]	1231	146871	0.04	0.07	0.1	0.14	0.2	0.27	0.35	0.43	0.52	0.61
O69	Labour and delivery complicated by umbilical cord complications	210	147892	0.03	0.04	0.05	0.06	0.07	0.08	0.09	0.11	0.13	0.15
O70	Perineal laceration during delivery	1969	146133	0.05	0.08	0.13	0.2	0.29	0.39	0.51	0.62	0.72	0.81
O71	Other obstetric trauma	69	148033	0.03	0.03	0.04	0.04	0.05	0.05	0.06	0.06	0.07	0.08
O72	Postpartum haemorrhage	502	147600	0.03	0.05	0.06	0.08	0.11	0.14	0.17	0.21	0.25	0.3
O73	Retained placenta and membranes, without haemorrhage	80	148022	0.03	0.03	0.04	0.04	0.05	0.05	0.06	0.07	0.07	0.08

O75	Other complications of labour and delivery, not elsewhere classified	273	147829	0.03	0.04	0.05	0.06	0.08	0.09	0.11	0.13	0.16	0.18
O80	Single spontaneous delivery	773	147329	0.04	0.05	0.08	0.11	0.14	0.19	0.24	0.29	0.36	0.43
O82	Single delivery by Caesarean section	192	147910	0.03	0.04	0.05	0.05	0.06	0.08	0.09	0.11	0.12	0.14
O86	Other puerperal infections	73	148029	0.03	0.03	0.04	0.04	0.05	0.05	0.06	0.06	0.07	0.08
O90	Complications of the puerperium, not elsewhere classified	77	148025	0.03	0.03	0.04	0.04	0.05	0.05	0.06	0.07	0.07	0.08
O99	Other maternal diseases classifiable elsewhere but complicating pregnancy, childbirth and the puerperium	641	147461	0.04	0.05	0.07	0.09	0.12	0.16	0.2	0.25	0.31	0.37
Q05	Spina bifida	86	148016	0.03	0.03	0.04	0.04	0.05	0.05	0.06	0.07	0.08	0.09
Q21	Congenital malformations of cardiac septa	379	147723	0.03	0.04	0.06	0.07	0.09	0.11	0.14	0.17	0.2	0.24
Q23	Congenital malformations of aortic and mitral valves	221	147881	0.03	0.04	0.05	0.06	0.07	0.08	0.1	0.12	0.13	0.16
Q24	Other congenital malformations of heart	114	147988	0.03	0.03	0.04	0.05	0.05	0.06	0.07	0.08	0.09	0.1
Q27	Other congenital malformations of peripheral vascular system	100	148002	0.03	0.03	0.04	0.04	0.05	0.06	0.07	0.07	0.08	0.09
Q38	Other congenital malformations of tongue, mouth and pharynx	160	147942	0.03	0.04	0.04	0.05	0.06	0.07	0.08	0.09	0.11	0.12
Q43	Other congenital malformations of intestine	182	147920	0.03	0.04	0.04	0.05	0.06	0.07	0.09	0.1	0.12	0.14
Q50	Congenital malformations of ovaries, Fallopian tubes and broad ligaments	98	148004	0.03	0.03	0.04	0.04	0.05	0.06	0.06	0.07	0.08	0.09
Q51	Congenital malformations of uterus and cervix	103	147999	0.03	0.03	0.04	0.04	0.05	0.06	0.07	0.07	0.08	0.1
Q60	Renal agenesis and other reduction defects of kidney	99	148003	0.03	0.03	0.04	0.04	0.05	0.06	0.06	0.07	0.08	0.09
Q61	Cystic kidney disease	236	147866	0.03	0.04	0.05	0.06	0.07	0.09	0.1	0.12	0.14	0.16

Q63	Other congenital malformations of kidney	172	147930	0.03	0.04	0.04	0.05	0.06	0.07	0.08	0.1	0.11	0.13
Q66	Congenital deformities of feet	149	147953	0.03	0.04	0.04	0.05	0.06	0.07	0.08	0.09	0.1	0.12
Q76	Congenital malformations of spine and bony thorax	113	147989	0.03	0.03	0.04	0.05	0.05	0.06	0.07	0.08	0.09	0.1
Q82	Other congenital malformations of skin	91	148011	0.03	0.03	0.04	0.04	0.05	0.06	0.06	0.07	0.08	0.09
Q85	Phakomatoses, not elsewhere classified	92	148010	0.03	0.03	0.04	0.04	0.05	0.06	0.06	0.07	0.08	0.09

Table S5. The association between PRS of IBS and common confounding factors. P value less than 0.05 represented significant association between IBS and common confounders. PRS: polygenic risk score; IBS: irritable bowel syndrome; SE: standard error, PC: principal component.

**Variables**	**Beta**	**SE**	***P***
Age (continuous)	-0.02771	0.01895	0.14
BMI (continuous)	0.004689	0.00243	0.05
PC1 (continuous)	0.008987	0.003875	0.02
PC2 (continuous)	-0.008113	0.003602	0.02
PC3 (continuous)	0.003603	0.003806	0.34
PC4 (continuous)	-0.046446	0.007081	5.42×10^{-11}
PC5 (continuous)	-0.04265	0.01598	0.007
PC6 (continuous)	0.007998	0.003952	0.04
PC7 (continuous)	-0.005764	0.004481	0.20
PC8 (continuous)	0.007863	0.004758	0.10
PC9 (continuous)	-0.064471	0.011260	1.03×10^{-8}
PC10 (continuous)	-0.022570	0.005468	3.66×10^{-5}
Gender (female/male)	-0.001921	0.004816	0.69
Smoking (Yes/No)	0.003616	0.004835	0.46
Alcohol (Yes/No)	0.01915	0.01417	0.18

Table S6. Individual-level MR results between IBS and identified outcomes in total and sex-stratified population. *P* value less than 0.05 represented significant causal association from IBS to disease outcomes in one-sample MR analysis. MR, mendelian randomization; IBS: irritable bowel syndrome; OR: odds ratio; CI: confidence interval; GERD: Gastro-oesophageal reflux disease; MR: mendelian randomization.

	**Total**		**Female**		**Male**	
**Descriptions**	**OR (95% CI)**	***P***	**OR (95% CI)**	***P***	**OR (95% CI)**	***P***
Depression	1.40 (1.21-1.63)	1.10×10^{-5}	1.62 (1.31-2.00)	8.31×10^{-6}	1.38 (0.94-2.00)	0.10
Oesophagitis	-	-	1.25 (1.06-1.47)	7.53×10^{-3}	-	-
GERD	1.28 (1.14-1.43)	2.55×10^{-5}	1.71 (1.44-2.03)	1.19×10^{-9}	0.88 (0.68-1.14)	0.33
Dyspepsia	1.57 (1.30-1.90)	3.23×10^{-6}	1.85 (1.41-2.43)	8.08×10^{-6}	1.48 (0.93-2.37)	0.10
Diaphragmatic hernia	1.28 (1.14-1.44)	2.11×10^{-5}	1.42 (1.19-1.69)	7.17×10^{-5}	1.10 (0.84-1.43)	0.49
Diverticular disease of intestine	1.33 (1.19-1.47)	2.03×10^{-7}	-	-	1.16 (0.91-1.47)	0.22

Table S7. Summary-level MR results between IBS and outcomes identified in PheWAS. MR, mendelian randomization; IBS, irritable bowel syndrome; PheWAS, phenome-wide association study. OR: odds ratio; CI: confidence interval; GERD: Gastro-oesophageal reflux disease; IVW: inverse-variance weighted.

**Descriptions**	**MR IVW (multiplicative random effects)**			**MR-Egger**			**Weighted median**		**Weighted mode**		**MR_PRESSO**				$P_{Steiger}$ [d]
	**OR (95% CI)**	***P***	$P_{heterogeneity}$ [a]	**OR (95% CI)**	***P***	$P_{pleiotropy}$ [b]	**OR (95% CI)**	***P***	**OR (95% CI)**	***P***	**OR (95% CI)**	***P***	$P_{distortion\ test}$ [c]	**Outlier Variants**	
Depression	1.07 (1.01-1.14)	0.02	0.06	1.00 (0.84-1.18)	0.94	0.35	1.08 (1.00-1.17)	0.05	1.11 (0.90-1.36)	0.33	1.09 (1.04-1.15)	1.77×10^{-3}			7.43×10^{-77}
GERD	1.09 (1.05-1.13)	3.72×10^{-5}	0.38	1.00 (0.89-1.12)	0.95	0.12	1.08 (1.01-1.14)	0.01	0.98 (0.84-1.16)	0.84	1.09 (1.05-1.13)	4.27×10^{-5}			8.75×10^{-79}
Dyspepsia	1.21 (1.13-1.30)	9.28×10^{-8}	0.20	1.07 (0.88-1.31)	0.50	0.22	1.19 (1.08-1.31)	5.32×10^{-4}	1.18 (0.92-1.53)	0.20	1.20 (1.12-1.28)	5.61×10^{-7}			3.77×10^{-72}
Diaphragmatic hernia	1.10 (1.05-1.15)	2.75×10^{-5}	0.05	1.13 (0.99-1.29)	0.07	0.67	1.11 (1.04-1.18)	0.002	1.13 (0.97-1.33)	0.12	1.10 (1.06-1.15)	1.21×10^{-5}	0.062	rs13321176; rs57810551;rs2731562	3.91×10^{-72}
Diverticular disease of intestine	1.13 (1.08-1.19)	3.00×10^{-6}	2.24×10^{-6}	1.03 (0.88-1.20)	0.72	0.19	1.09 (1.02-1.15)	0.006	1.07 (0.94-1.22)	0.33	1.11 (1.07-1.16)	5.23×10^{-6}			3.87×10^{-56}

[a] *P* values for heterogeneity were derived from heterogeneity test and a *P* value < 0.05 indicates a possible heterogeneity.

[b] *P* values for pleiotropy were derived from MR-Egger test and a *P* value < 0.05 indicates a possible pleiotropic effect.

[c] *P* values for distortion were derived from MR-PRESSO test and a *P* value < 0.05 indicates a difference between estimates before and after outlier removal.

[d] *P* values for steiger were derived from direction test and a *P* value < 0.05 indicates correct direction.

P value less than 0.05 represented significant causal association from IBS to disease outcomes in summary-level MR analysis.

Table S8. Identified pleiotropic SNPs of the association between genetically predicted IBS and disease outcomes with the significance threshold FDR=0.05 in the PheWAS analysis. SNP: single nucleotide polymorphism; IBS: irritable bowel syndrome; FDR: false discovery rate; PheWAS: Phenome-wide association study; SE: standard error; OR: odds ratio.

Pleiotropic SNP	Beta	SE	OR	*P*	Phenotype
rs13321176	-0.04289667	0.010100004	0.96 (0.94-0.98)	2.16×10^{-5}	Diverticular disease
rs2731562	0.07508206	0.007380233	1.08 (1.06-1.09)	2.61×10^{-24}	Diverticular disease
rs57810551	0.03901330	0.007681037	1.04 (1.02-1.06)	3.79×10^{-7}	Diverticular disease

Table S9. Sensitivity analysis of MR results by using additional publicly available GWAS summary data. IBS: irritable bowel syndrome; OR: odds ratio; CI: confidence interval; GERD: Gastro-oesophageal reflux disease; IVW: inverse-variance weighted; MR: mendelian randomization.

Descriptions	MR IVW (multiplicative random effects)			MR-Egger			Weighted median		Weighted mode		MR_PRESSO				
	**OR (95% CI)**	***P***	***P* heterogeneity [a]**	**OR (95% CI)**	***P***	***P* pleiotropy [b]**	**OR (95% CI)**	***P***	**OR (95% CI)**	***P***	**OR (95% CI)**	***P***	***P* distortion test [c]**	**Outlier Variants**	***P* Steiger [d]**
Depression	1.14 (1.10-1.18)	3.68×10^{-14}	1.75×10^{-11}	1.03 (0.93-1.14)	0.56	0.04	1.08 (1.04-1.12)	3.22×10^{-5}	1.07 (0.98-1.16)	0.13	1.12 (1.09-1.16)	7.01×10^{-10}	-	-	1.47×10^{-100}
GERD	1.20 (1.16-1.24)	2.77×10^{-26}	3.20×10^{-4}	1.11 (1.01-1.23)	0.04	0.13	1.19 (1.14-1.24)	1.36×10^{-16}	1.26 (1.13-1.42)	1.00×10^{-4}	1.19 (1.13-1.24)	1.75×10^{-8}	-	-	1.98×10^{-59}
Dyspepsia	1.03 (1.02-1.04)	5.07×10^{-8}	0.84	1.03 (0.99-1.07)	0.19	0.86	1.03 (1.01-1.05)	2.00×10^{-4}	1.03 (1.00-1.07)	0.09	1.03 (1.02-1.04)	9.75×10^{-6}	-	-	6.19×10^{-85}
Diaphragmatic hernia	1.14 (1.08-1.20)	4.87×10^{-7}	3.00×10^{-4}	1.16 (0.99-1.37)	0.08	0.84	1.17 (1.09-1.24)	3.73×10^{-6}	1.21 (1.01-1.44)	0.04	1.12 (1.06-1.19)	1.46×10^{-4}	-	-	1.34×10^{-80}

Diverticular disease of intestine	1.01 (1.01-1.02)	4.12×10^{-7}	1.00 (0.99-1.01)	0.72	0.04	1.01 (1.00-1.01)	6.45×10^{-5}	1.01 (1.00-1.01)	0.13	1.01 (1.01-1.01)	1.17×10^{-5}	0.317	rs13321176;rs57810551	1.48×10^{-52}

[a] *P* values for heterogeneity were derived from heterogeneity test and a *P* value < 0.05 indicates a possible heterogeneity.

[b] *P* values for pleiotropy were derived from MR-Egger test and a *P* value < 0.05 indicates a possible pleiotropic effect.

[c] *P* values for distortion were derived from MR-PRESSO test and a *P* value < 0.05 indicates a difference between estimates before and after outlier removal.

[d] *P* values for steiger were derived from direction test and a *P* value < 0.05 indicates correct direction.

P value less than 0.05 represented significant causal association from IBS to disease outcomes in summary-level MR analysis.

Table S10. Bidirectional MR results with 5 identified disease conditions as exposure and IBS as outcome. IBS: irritable bowel syndrome; OR: odds ratio; CI: confidence interval; GERD: Gastro-oesophageal reflux disease; IVW: inverse-variance weighted; MR: mendelian randomization;

		**MR IVW (multiplicative random effects)**			**MR-Egger**			**Weighted median**		**Weighted mode**		**MR PRESSO**			
**Exposures**	**Number of snps**	**OR (95% CI)**	***P***	***P* heterogeneity [a]**	**OR (95% CI)**	***P***	***P* pleiotropy [b]**	**OR (95% CI)**	***P***	**OR (95% CI)**	***P***	**OR (95% CI)**	***P***	***P* distortion test [c]**	**Outlier Variants**
Depression	209	1.26 (1.22-1.31)	1.46×10^{-37}	1.25×10^{-5}	1.16 (1.01-1.33)	0.04	0.23	1.21 (1.15-1.26)	6.21×10^{-16}	1.19 (1.05-1.36)	0.01	1.26 (1.21-1.30)	5.15×10^{-28}	0.83	rs3099439
GERD	128	1.27 (1.11-1.45)	4.43×10^{-4}	0.05	1.38 (1.11-1.71)	0.005	0.36	1.29 (1.04-1.59)	0.02	1.28 (0.12-13.78)	0.84	1.27 (1.11-1.45)	6.10×10^{-4}		
Dyspepsia	18	1.00 (1.00-1.00)	0.71	0.74	1.00 (1.00-1.00)	0.08	0.04	1.00 (1.00-1.00)	0.92	1.00 (1.00-1.00)	0.85	1.00 (1.00-1.00)	0.72		
Diaphragmatic hernia	42	1.06 (1.01-1.11)	0.01	3.10×10^{-4}	0.95 (0.81-1.11)	0.53	0.17	1.03 (0.98-1.08)	0.31	1.00 (0.90-1.11)	0.99	1.05 (1.01-1.09)	0.03	0.52	rs2523598
Diverticular disease of intestine	116	3.99 (2.83-5.62)	2.89×10^{-15}	0.03	1.51 (0.55-4.14)	0.42	0.05	3.03 (1.85-4.97)	1.12×10^{-5}	3.45 (1.27-9.34)	0.02	3.54 (2.60-4.82)	1.14×10^{-12}	0.44	rs1388625;rs17730380

[a] *P* values for heterogeneity were derived from heterogeneity test and a *P* value < 0.05 indicates a possible heterogeneity.

[b] *P* values for pleiotropy were derived from MR-Egger test and a *P* value < 0.05 indicates a possible pleiotropic effect.

[c] *P* values for distortion were derived from MR-PRESSO test and a *P* value < 0.05 indicates a difference between estimates before and after outlier removal.

P value less than 0.05 represented significant causal association from IBS to disease outcomes in summary-level MR analysis.

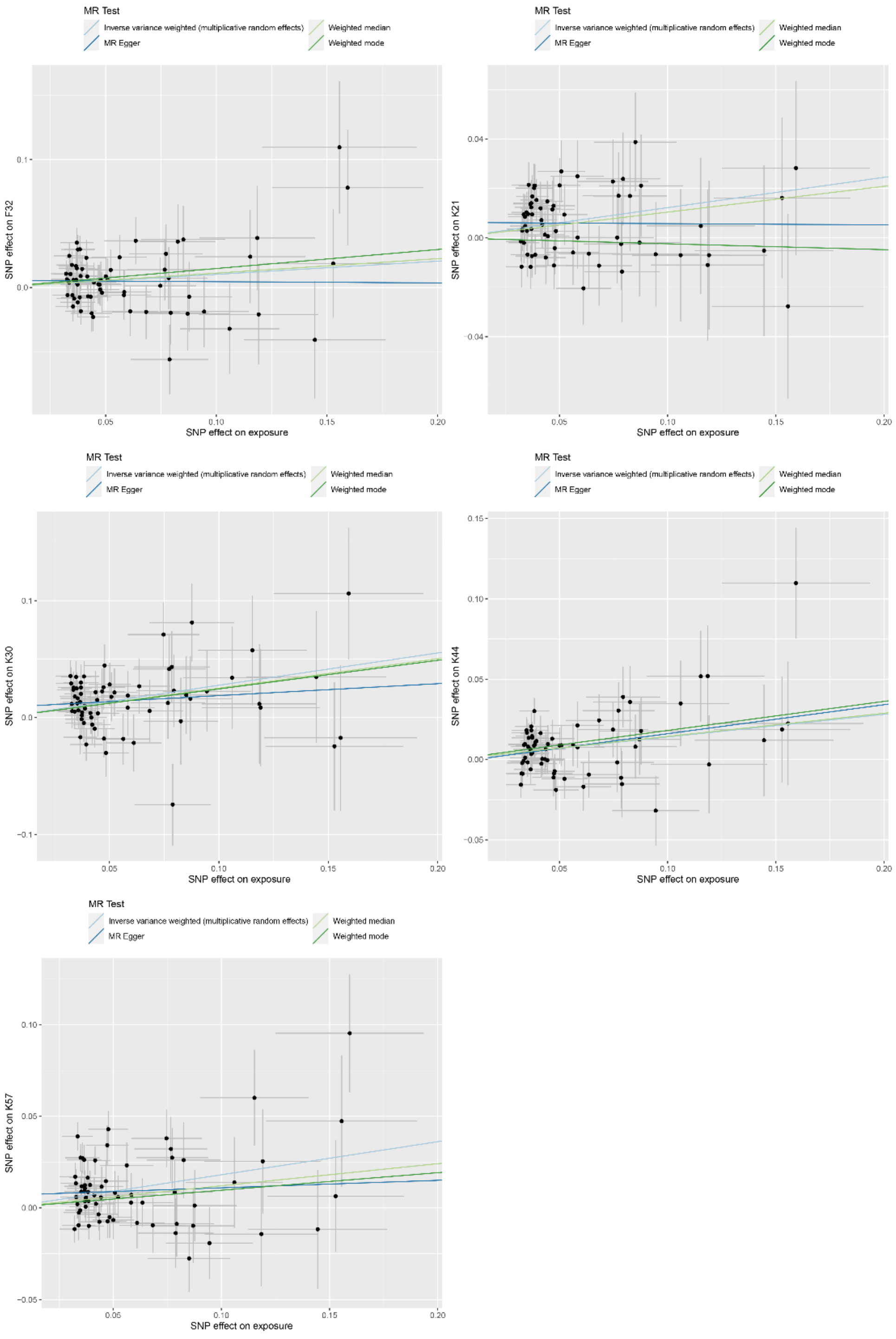

Figure S1. Scatter plots to visualize the causal effect between IBS and depression, GERD, diaphragmatic hernia, dyspepsia and diverticular disease. The slope of the straight lines correspond to causal estimates using each of four different methods (IVW random effects, MR-Egger, weighted median, and weighted mode). Outlier SNPs are labeled. IBS, irritable bowel syndrome; GERD, gastroesophageal reflux disease; MR, Mendelian randomization; SNP, single nucleotide polymorphism; IVW, inverse-variance weighted.

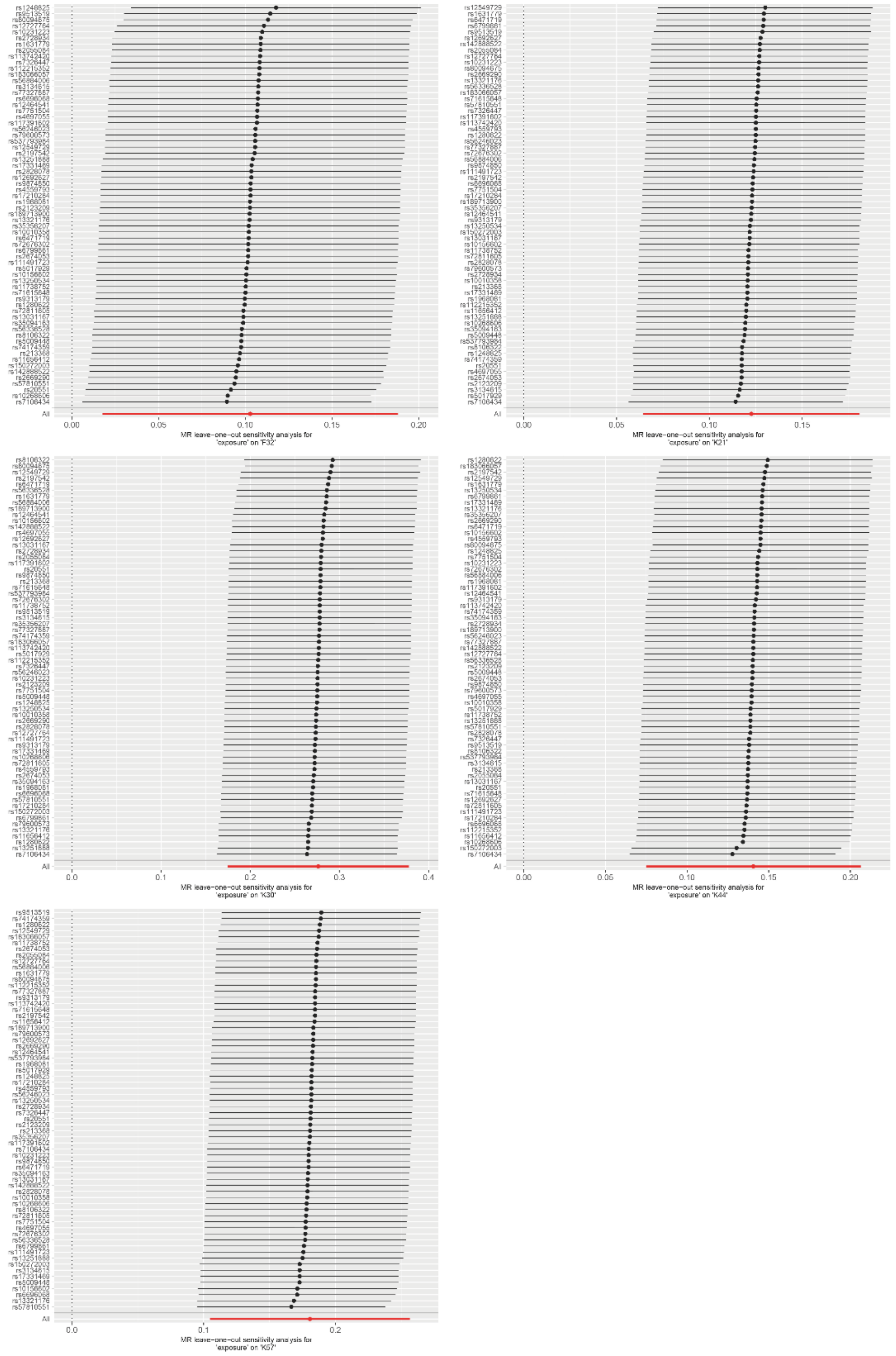

Figure S2. Leave-one-out sensitivity analysis of the causal associations between IBS and depression, GERD, diaphragmatic hernia, dyspepsia and diverticular disease. Each black point represents the effect size in the causal correlation of IBS on outcomes excluding that particular variant from the analysis. The red point depicts the estimated effect sizes using all SNPs. IBS, irritable bowel syndrome; GERD, gastroesophageal reflux disease; SNP, single nucleotide polymorphism.
