# Supplementary material for: The Causal Association of Irritable Bowel Syndrome with Multiple Disease Outcomes: A Phenome-Wide Mendelian Randomization Study
Source: J Clin Med. 2023 Jan 31;12(3):1106. doi: 10.3390/jcm12031106 (PMC9918111; doi:10.3390/jcm12031106)
Supplement: Supplementary file 1 [file jcm-12-01106-s001.zip › Supplementary Methods.pdf]

Supplementary Materials

1. Definitions of irritable bowel syndrome (IBS) cases

IBS cases in the UK Biobank were defined as meeting at least one of the following four criteria:

Digestive Health Questionnaire (DHQ)-based Roma III definition

1) The DHQ was incorporated into the UK Biobank questionnaire platform and advertised via email, which recorded on-line gastro-intestinal health self-assessment questionnaire issued in 2017.

Participants who completed the DHQ and whose abdominal symptoms were consistent with the diagnosis of IBS based on the following four Roma III definitions.

Criteria 1: Frequency of discomfort/pain in abdomen in last 3 months was evaluated through the question "In the last 3 months, how often did you have discomfort or pain anywhere in your abdomen?", we classified as "Two to three days a month", "One day a week", "More than one day a week", "Every day" as "yes".

Criteria 2: Discomfort/pain occurring only during menstrual bleed was evaluated through the question "Did this discomfort or pain occur only during your menstrual bleeding and not at other times?", we categorized as "no" or "yes", and all the male participants as "yes".

Criteria 3: Abdominal discomfort/pain for 6 months or longer was ascertained through the question "Have you had this discomfort or pain 6 months or longer?", the answer "yes" was assigned positive answer.

Criteria 4: defined as meeting at least two of these following:

Number	Descriptions	Questions	Positive answers in UK Biobank
1	Frequency of discomfort/pain getting better or stopping after a bowel movement	"How often did this discomfort or pain get better or stop after you had a bowel movement?"	"Sometimes", "Often", "Most of the time", "Always"
2	More frequent bowel movements when abdominal discomfort/pain started	"When this discomfort or pain started, did you have more frequent bowel movements?"	"Sometimes", "Often", "Most of the time", "Always"
	OR Less frequent bowel movements when abdominal discomfort/pain started	"When this discomfort or pain started, did you have less frequent bowel movements?"	"Sometimes", "Often", "Most of the time", "Always"
3	Stools looser when abdominal discomfort/pain started	"When this discomfort or pain started, were your stools (bowel movements) looser?"	"Sometimes", "Often", "Most of the time", "Always"
	OR Frequency of harder stools when abdominal discomfort/pain started	"When this discomfort or pain started, how often did you have harder stools?"	"Sometimes", "Often", "Most of the time", "Always"

2) DHQ-based prompted self-reported previous IBS diagnosis

Participants who have ever been diagnosed as IBS was ascertained by the question “Have you ever been diagnosed with IBS?”, the answer yes was defined as positive answer.

3) Unprompted self-reported previous IBS diagnosis

Previous unprompted self-reported diagnosis of IBS was answered by the question “serious medical conditions previously diagnosed by a doctor” at recruitment visit (2006-2010), and in subsequent UK Biobank clinic follow-ups in 2012-2013, 2014 and 2019. Participants who reported previous diagnosis of IBS (UKB coding of 1154) was defined as “yes”. The definition of IBS was defined as the If the participant was uncertain of the type of illness they had had, then they described it to the interviewer (a trained nurse) who attempted to place it within the coding tree.

4) ICD-10 diagnosis of hospital admission

Participants who diagnosed with IBS (main or secondary ICD-10 codes as K58) at the initial assessment or follow-up visit were defined as IBS cases.

5) Excluding criteria of IBS cases

Participants who met Roma III criteria but had other confounding conditions before IBS diagnosis were excluded from both cases and controls (excluding criteria was shown in Table S1).

2. Genome-wide association study

Prior to PheWAS, we performed Genome-wide association study (GWAS) for IBS in base data and calculated the polygenic risk score (PRS) for each participant as a proxy of genetic liability for IBS in target data using this GWAS summary statistics. Genotyping in the UK Biobank was performed using the UK BiLEVE AxiomTM Array and UK Biobank Axiom array from Affymetrix, and variants were imputed using the haplotype reference consortium, as well as merged UK10K and 1000 Genomes phase 3 reference panels [45].

We first performed a standard quality control procedure at the SNP level as follows: SNPs with call rate >99% (less than 1% missing data), MAF>1%, and Hardy–Weinberg equilibrium (HWE)

$P < 1.0 \times 10^{-6}$ [46], which resulted in a total of 6,279,351 SNPs and 173,176 individuals in base data retained for GWAS analysis. Each variant was coded as 0, 1, or 2, according to the number of effect alleles. Then, a generalized linear mixed model (GLMM), controlling for population stratification and relatedness, was adopted for the GWAS [18].

3. Ascertainment of polygenic risk score of IBS

Individual polygenic risk score (PRS) in target data were calculated according to the tutorial [17]. In brief, palindromic single nucleotide polymorphism (SNP) (i.e., SNPs with A/T or C/G at intermediate frequencies) were firstly removed to select valid SNPs for PRS [17,47]. In order to find "best-fitted" PRS of IBS, we tested different sets of pruned (r^2<0.1, and in the 250 kb region) SNPs passing with different p value thresholds (0.00001, 0.0001, 0.001, 0.01, 0.05, 0.1, 0.2, 0.3, 0.4, and 0.5), by using logistic regression adjusted with age (continuous variables), gender (male/female) and principal components (PC) 1 to 20 (continuous variables) [17]. The performances of different models were listed as follows:

Threshold of P	Number of SNPs used in PRS	R^2 of model	P	OR (95%CI)
0.000001	2	9.87e-06	0.268493	1.01 (0.99-1.02)
0.00001	15	1.33e-05	0.200164	1.01 (0.99-1.03)
0.0001	163	0.00013424	4.60×10^{-5}	1.03 (1.02-1.05)
0.001	1182	0.00035937	2.63×10^{-11}	1.05 (1.04-1.07)
0.01	8697	0.00066147	1.55×10^{-19}	1.07 (1.06-1.09)
0.05	33488	0.00110886	1.25×10^{-31}	1.09 (1.08-1.11)
0.1	58314	0.00118792	9.22×10^{-34}	1.10 (1.08-1.11)
0.2	99648	0.00118271	1.27×10^{-33}	1.10 (1.08-1.11)
0.3	134047	0.00118733	9.57×10^{-34}	1.10 (1.08-1.11)
0.4	164050	0.00113851	1.98×10^{-32}	1.09 (1.08-1.11)
0.5	190077	0.00114793	1.10×10^{-32}	1.09 (1.08-1.11)

We found that PRS with a p threshold of 0.1 explained the highest phenotypic variance of 0.12%. Finally, 58,314 SNPs were retained for PRS calculation by summing the reference alleles for each SNP weighted by the summary regression coefficient for IBS. The PRS was calculated using PLINK1.9 software [17].

Subsequently, we performed linear or logistic regressions to ensure that the PRS of IBS was not associated with other common confounders of health outcomes, including age (continuous variable) [48], sex (female/male) [48], body mass index (BMI) (continuous variable) [49], genetic primary components (continuous variable), smoking status (yes/no) [50], and alcohol status (yes/no) [51].

4. Ascertainment of sex-specific PRS of IBS

Sex-specific GWAS of IBS was firstly analyzed in base data in female and male, separately. Then, sex-specific PRS of IBS in target data was calculated based on the GWAS summary statistics derived from female and male in base, separately. As mentioned above, we tested different sets of pruned (r^2<0.1, and in the 250 kb region) SNPs passing with different p value thresholds (0.00001, 0.0001, 0.001, 0.01, 0.05, 0.1, 0.2, 0.3, 0.4, and 0.5), by using logistic regression adjusted with age (continuous variables) and principal components 1 to 20 (continuous variables) [17]. The performances of different models in female and male were listed as follows:

**Models in female**				
Threshold of P	Number of SNPs used in PRS	R^2 of model	P	OR (95%CI)
0.000001	2	2.95e-06	0.629325	1.00 (0.99-1.02)
0.00001	13	6.71e-05	0.021348	1.02 (1.00-1.04)
0.0001	130	0.000162	0.000356	1.03 (1.01-1.05)
0.001	1097	0.00032	5.02×10^{-7}	1.05 (1.03-1.07)
0.01	8533	0.000809	1.41×10^{-15}	1.07 (1.06-1.10)
0.05	33105	0.000919	1.72×10^{-17}	1.08 (1.06-1.10)
0.1	57954	0.000944	6.53×10^{-18}	1.08 (1.06-1.10)
0.2	99396	0.000764	8.47×10^{-15}	1.07 (1.05-1.09)
0.3	134220	0.000824	7.76×10^{-16}	1.08 (1.06-1.10)
0.4	163847	0.000854	2.36×10^{-16}	1.08 (1.06-1.10)
0.5	189834	0.000845	3.31×10^{-16}	1.08 (1.06-1.10)
**Models in male**				
Threshold of P	Number of SNPs used in PRS	R^2 of model	P	OR (95%CI)

0.000001	2	1.72e-05	0.397011	1.01 (0.98-1.04)
0.00001	13	8.03e-06	0.561972	0.99 (0.97-1.02)
0.0001	115	2.72e-05	0.285642	0.99 (0.96-1.01)
0.001	976	1.72e-05	0.39628	1.01 (0.98-1.04)
0.01	7991	0.000159	0.009947	1.04 (1.01-1.06)
0.05	31708	0.000332	0.000192	1.05 (1.02-1.08)
0.1	56630	0.00032	0.000251	1.05 (1.02-1.08)
0.2	98057	0.000543	1.84×10^{-6}	1.07 (1.04-1.10)
0.3	132965	0.000543	1.85×10^{-6}	1.07 (1.04-1.10)
0.4	163411	0.000547	1.71×10^{-6}	1.07 (1.04-1.10)
0.5	189590	0.000533	2.32×10^{-6}	1.07 (1.04-1.10)

We found that PRS with a p threshold of 0.1 in female and 0.4 in male explained the highest phenotypic variance of 0.09% and 0.05%, respectively. Finally, 57,954 SNPs for female and 163,411 SNPs for male were retained for PRS calculation. The PRS was calculated using PLINK1.9 software [17].

5. GWAS summary statistics from publicly available study

We chose genetic instruments from a previously published study with a much larger sample size to increase statistical power [52]. Briefly, this study analyzed 53,400 IBS cases and 433,201 controls from the UK Biobank and Bellygenes initiative using a linear mixed model adjusted for age, sex, age × sex, sex × age^2, and the first 20 PCs of genetic data) [9].

In order to replicate our findings on the association between IBS and disease outcomes identified in PheWAS, publicly available summary-level data for depression [53], gastro-oesophageal reflux disease (GERD) [54], dyspepsia [55], diaphragmatic hernia [56], and diverticular disease [57] were obtained. Briefly, GWAS summary-level data of depression was obtained from one study containing 807,553 individuals (246,363 cases and 561,190 controls) from three largest genome-wide association studies, the UK Biobank, 23andMe, and Psychiatric Genomics Consortium (PCG) cohort [53]. A GWAS of GERD was obtained from a meta-analysis of 385,276 participants (80,265 cases, 305,011 controls) containing data from the UK Biobank, 23adMe, and QSkin studies [54]. A GWAS of dyspepsia was derived from a study containing 361,360

individuals (10,078 cases and 351,282 controls) combining four study cohorts of UK Biobank, EGCUT, MGI, and Translational Research Center for Gastrointestinal Disorder (TARGID) [55]. A GWAS of diaphragmatic hernia was obtained from one study containing 374,296 individuals (31,193 cases and 343,103 controls) from the UK Biobank [56]. A GWAS of diverticular disease was derived from a study containing 409,728 participants (27,444 cases; 382,284 controls) in the UK Biobank [57].
